# Cell-Laden Supramolecular and Covalent Polymer Hydrogels for High-Shear Delivery: A Design of Experiments Approach

**DOI:** 10.1021/acs.chemmater.5c03073

**Published:** 2026-02-16

**Authors:** Penelope E. Jankoski, Jessica Shrestha, Windfield S. Swetman, Harrison Livingston, Jamie Sorrell, Tristan D. Clemons

**Affiliations:** † School of Polymer Science and Engineering, 5104 University of Southern Mississippi, Hattiesburg, Mississippi 39406, United States; ‡ Sumrall High School, Sumrall, Mississippi 39402, United States

## Abstract

Effective design of cell-delivery scaffolds is of key importance for regenerative medicine technologies to meet their full potential, especially when considering cell delivery to wounds of complex architecture or directly into the biological environment. Few studies, however, focus on a systematic approach to understanding the cell, polymer scaffold, and final biomaterial properties of this composite material. In this work, we report on the systematic analysis of a supramolecular hydrogel composed of ionically cross-linked peptide amphiphile (PA) nanofibers, optimized for high-shear delivery of therapeutic cells, and compare the performance of this biomaterial to a covalent polymer hydrogel of ionically cross-linked alginate. Using a full factorial design of experiments (DoE), we investigated the interplay between polymer concentration and cell loading to determine the impact on mechanical properties, structural integrity, substrate adhesion, and sprayability of the hydrogel. The shear-thinning and thixotropic nature of the supramolecular hydrogels enabled effective deposition through a spray nozzle, not possible with the alginate hydrogel, while preserving cell viability and hydrogel mechanical properties. The supramolecular backbone of the PA nanofibers enabled remarkable mechanical resilience and full recovery post-spray, even at cell loadings as high as 2 million cells/mL, while significant loss of gel integrity was observed with the alginate hydrogel at equivalent cell loadings. Our findings establish a robust structure–property relationship framework for the formulation of cell-laden supramolecular hydrogels capable of high-shear delivery, highlighting their potential as customizable platforms for regenerative medicine, advanced wound care, and 3D printing applications.

## Graphical abstract

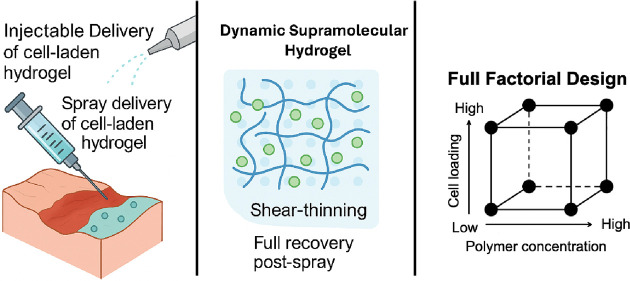

## Introduction

The delivery of living cells underpins many emerging strategies in regenerative medicine, yet transporting fragile cells into target tissues remains a formidable challenge.^1–4^ Approaches such as injection, spray deposition, and extrusion-based bioprinting promise minimally invasive delivery with precise spatial control but expose both the polymeric hydrogel scaffolds and the embedded cells to extreme mechanical stresses. The selection of polymeric carrier for the high-shear delivery of cells is a multifaceted challenge, as materials must possess both biocompatibility and appropriate rheological properties to maintain structural integrity through deposition.^5,6^ The interplay between viscosity, gel stiffness, shear-thinning behavior, and cross-linking kinetics are critical parameters to consider for injection, extrusion, or spray delivery of these hydrogel materials.^7–12^ A sol–gel transition through shear enables these delivery modalities, governed by the ability of the biomaterial to disaggregate and flow through shear and quickly reform a gel following deposition.^13,14^ Moreover, the ability to undergo rapid in situ gelation is paramount for preserving the scaffold microarchitecture and functionality of the hydrogel to support cell localization and regenerative potential.^14,15^ As a result, maintaining material integrity and cell viability under high shear has become a central barrier to the translation of these regeneration approaches. Despite the rapid growth of cell-based therapies, there remains a critical gap in understanding how the interplay between hydrogel mechanics and cell loading governs performance during high-shear delivery.^5,6,16–21^ Design strategies that couple tunable yield stress with self-healing kinetics provide a route to hydrogels that are both highly injectable and mechanically resilient, meeting the dual demands of delivery and long-term function in vivo.^8,22–25^

The delivery of cells within a host material, or cell-laden hydrogels, has been an area of recent interest, as it provides the opportunity to deliver cells within a stable platform to the site of injury or need.^26–29^ 3D printing of polymeric bioinks that integrate cells has been of specific interest, as it enables specification of extracellular features and cellular organization for increased control over tissue fabrication strategies and the emerging field of organoid development.^30–34^ Despite this excitement, the majority of these studies and others in the field focus solely on the biological outcome of cellular delivery and tissue regeneration without investigating the impact on fundamental material properties. When loading the matrix with high volumes of cells or filler when considering these materials as polymer composites, it is expected that gel stiffness and other properties that depend on network integrity would diminish as a result of filler aggregation disrupting the uniformity of the matrix.^35–39^ Cells, due to their nonlinear mechanical characteristics and bulk, can disrupt the cross-linking and in turn the structural integrity of the hydrogel network, leading to decreased stiffness and altered rheological behavior.^38,40^

The high-shear delivery of cell-laden hydrogels via spray and 3D bioprinting is an emerging field, but the effects of high-shear forces on both material integrity with maintained cell function remain insufficiently understood.^41–43^ Spray deposition and extrusion-based printing both subject these biomaterials to high shear, but the demands differ: spray delivery prioritizes atomization and rapid in situ reassembly across irregular surfaces, while extrusion requires filament stability and pattern fidelity. In both cases, materials must fluidize under shear yet swiftly recover to protect cells and preserve the geometry. Spray delivery provides a practical means to apply cells and matrices to extensive or irregular wounds with minimal invasiveness. Sprayable fibrin–keratinocyte suspensions have demonstrated feasibility by enhancing cell retention and wound coverage, but such systems are primarily adhesive and lack the structural and bioactive complexity required to direct long-term regeneration.^44–49^ Similarly, extrusion-based 3D printing of hydrogel bioinks subject cell-laden formulations to high-shear environments, necessitating rheologically engineered networks that can transiently fluidize for deposition while rapidly recovering structural integrity to preserve cell viability and spatial fidelity.^50–55^ When viscosity, shear-thinning response, yield stress, or gelation/recovery times fall outside the processing window, materials are prone to nozzle clogging, filament collapse, loss of pattern fidelity, and reduced cell viability, outcomes repeatedly noted across extrusion and related hydrogel printing modalities.^28,55–58^ These constraints do not merely affect printability; they govern biological performance by dictating whether spatial cues, mechanical support, and payload localization are preserved post-deployment.^27,57^ As noted by Bertsch et al., self-healing injectable hydrogels are at the forefront of regenerative medicine because the ability to fluidize under shear and subsequently recover enables minimally invasive, patient-specific delivery and sustained support for tissue repair.^59^ Consequently, next-generation matrices should be engineered with processing-aware rheology, combining pronounced shear-thinning with fast self-recovery and tunable mechanics so that deposition and function are optimized together.

Peptide amphiphile (PA) supramolecular polymers and alginate represent two distinct hydrogel design paradigms that offer complementary opportunities for cell delivery under high shear. Supramolecular polymers of PAs self-assemble into nanofibers through hydrophobic collapse, beta-sheet hydrogen bonding, and electrostatic interactions between monomers.^60–62^ Their noncovalent assembly and ionically mediated cross-linking impart dynamic, shear-thinning hydrogels that fluidize under stress yet rapidly reform upon cessation, enabling minimally invasive injection or spray delivery. This dynamic property renders them ideal candidates for spray or injection delivery applications, facilitating minimally invasive administration following high shear.^63–65^ These hydrogels exhibit tunable mechanical properties and have found diverse applications, ranging from inks for 3D bioprinting,^66,67^ to scaffolds for tissue regeneration.^61,68,69^ In contrast, alginate is a covalently linked polysaccharide that forms ionically cross-linked networks through divalent coordination.^70–74^ Alginate is a linear polysaccharide of 1→4 linked beta-D-mannuronic acid (M) and α-L-guluronic acid (G) residues arranged in homopolymeric M- and G-blocks as well as alternating sequences. The block composition dictates ion binding and gel architecture: contiguous G-blocks adopt a rigid conformation that coordinates divalent cations in cooperative ‘egg-box’ junctions, producing dense cross-linking zones and mechanically robust networks, whereas the more flexible M-blocks bind cations weakly, promoting increased swelling and softer gels.^75,76^ Alginate hydrogels have been extensively applied in tissue regeneration and 3D bioprinting, making it a widely used benchmark material.^30,38,70^ Despite this, few studies have systematically examined how gel properties of either system are altered by high cell loading and an ionic environment. Direct comparison of PAs and alginate thus provides a unique opportunity to uncover structure–property relationships as a result of backbone chemistry that governs the performance of cell-laden hydrogels affecting their efficacy through high-shear delivery.

The goal of this work was to develop an understanding of the interactions between matrix concentration and cell concentration that impact biomaterial properties while maintaining cell viability through high-shear delivery. To identify variable interactions and influencing factors, a Quality by Design process can be implemented using a full factorial Design of Experiments (DoE) approach. A full factorial design is an experimental approach that examines combinations of multiple factors and their levels, allowing for a better understanding of the full design space.^77,78^ Within a DoE framework, a factor represents an experimental variable under investigation (e.g., concentration, pH, temperature), while a level corresponds to the specific values or conditions under which that factor is tested. This methodology is crucial for understanding interactions between variables, as it allows observation of how one factor’s effect may depend on another’s level. Identifying such interactions is essential because they can reveal synergistic or antagonistic effects that might not be apparent when factors are studied in isolation.^79,80^

Despite the widespread use of covalent and supramolecular hydrogels in therapeutic cell delivery, there has been little direct comparison of how these polymeric materials accommodate high cell loads. Covalent polymers, such as alginate, form stable ionically cross-linked networks, whereas PAs assemble through noncovalent interactions into highly dynamic supramolecular matrices. These divergent paradigms are expected to yield distinct rheological responses to cell loading, yet this has not been systematically examined. Here, we apply a full factorial DoE framework to compare alginate and PA supramolecular polymers across matrix and cell concentrations, establishing structure–property relationships that reveal how material chemistry governs injectability, recovery, and integrity. These insights provide design rules for optimizing cell-laden hydrogels for applications in sprayable and injectable delivery platforms for future tissue regeneration applications.

## Materials and Methods

### Materials

Acetonitrile, dichloromethane (DCM), *N*,*N*-diisopropylethylamine (DIEA), *N*,*N*-dimethylformamide (DMF), diethyl ether, methanol, 4-methylpiperidine, and sodium hydroxide (NaOH) were all purchased from Sigma-Aldrich and used as received. Palmitic acid was obtained from Acros Organics and used as received. All Fmoc-protected amino acids, Oxyma, and Rink amide MBHA resin were purchased from CEM. Triisopropylsilane (TIPS), trifluoroacetic acid (TFA), Dulbecco’s modified eagle medium (DMEM), heat-inactivated fetal bovine serum (FBS), and penicillin-streptomycin, the CyQUANT lactate dehydrogenase (LDH) assay, and Live/Dead Imaging kits were all purchased from ThermoFisher. Sterile, tissue-treated 6- and 96-well plates were obtained from CellTreat (USA). Sodium alginate was obtained from MPBio with 95% purity. All other chemicals were purchased from ThermoFisher. Bacon fat and chicken drumsticks were obtained from Winn Dixie.

### Peptide Amphiphile (PA) Synthesis

The PA used in this study (peptide sequence C_16_V_3_A_3_E_3_) was synthesized using standard solid-phase Fmoc peptide chemistry. PAs were synthesized on Rink amide MBHA resin (EMD) with the amino acid couplings performed on a CEM Liberty Blue microwave-assisted peptide synthesizer (CEM, Matthews, NC, USA). Fmoc groups were cleaved using 20% 4-methylpiperidine in *N*,*N*-dimethylformamide (DMF) at 90 °C for 30 s. Amino acids were coupled using 4 mol equivalents (equiv) of each Fmoc-protected amino acid, 8 equiv ethyl cyanohydroxyiminoacetate (Oxyma), and 8 equiv of *N*,*N*′-diisopropylcarbodiimide (DIC) for 2–4 min at 90 °C in 50:50 DMF as solvent. Using this same procedure, palmitic acid (C_16_) was conjugated to the N-terminus of the peptide as the hydrophobic tail.

Completed PA molecules were cleaved off the resin using a solution of 95:2.5:2.5 trifluoroacetic acid (TFA)/triisopropylsilane (TIPS)/water for 2–3 h at ambient temperature on a wrist shaker. Following cleavage, PAs were precipitated with cold diethyl ether, collected via centrifugation, and dried overnight. The PAs were then purified by preparative-scale reverse phase high-performance liquid chromatography (CEM Prodigy HPLC) using a Phenomenex Gemini column (C-18 stationary phase, 5 μm, 100 Å pore size, 50 × 250 mm). A mobile phase of acetonitrile and water was used, both containing 0.1% ammonium hydroxide. Pure fractions were identified using electrospray ionization mass spectroscopy (ESI-MS) in negative ion mode on a ThermoScientific Orbitrap ExplorisTM 240 mass spectrometer using direct injection. Excess acetonitrile was removed with rotary evaporation and freeze-dried, and the powders were stored at −20 °C until use.

### Liquid Chromatography Mass Spectrometry (LC-MS)

The purity of the synthesized PA was confirmed using liquid chromatography-mass spectroscopy (LC-MS), which was performed using an Agilent 1200 system with a Phenomenex Gemini C-18 column (100 × 1.00 mm; 5 μm) for basic conditions. The mass detector (MS) was an Agilent 6520 Q-TOF MS. All gradient methods followed: acetonitrile at 5% for 5 min at 50 μL/min, 5–95% over 25 min at 50 μL/min, followed by 95% for 5 min at 50 μL/min. Ammonium hydroxide (0.1% v/v) for basic conditions was added to all solvents. Peaks were detected at λ = 220 nm.

### Peptide Amphiphile Preparation

Lyophilized powder was reconstituted in deionized water to a concentration of 1 wt %, and the pH was adjusted to 7.4 using NaOH. The solution was then aliquoted, lyophilized, and stored at −20 °C until further use. For the preparation of cell-laden hydrogels, polymer samples were dissolved at twice the desired final concentration and mixed volumetrically (1:1) with a gelling solution containing calcium and cells at 2× their target concentrations. Upon thorough mixing, the resulting hydrogels reached the intended final polymer, calcium, and cell concentrations. Samples were allowed to gel for 15 min at room temperature prior to subsequent use.

### Calcium Quantification

Hydrogels (0.25, 0.5, and 1.0 wt %, 100 μL) were prepared in wells in a 96-well plate as described above and allowed to ionically cross-link. Phosphate buffered saline (PBS) sink (100 μL) was added, and the samples were incubated at 37 °C with the sink collected and exchanged at defined time points (0–72 h). Aliquots were frozen at −20 °C until completion of the kinetics, thawed, and then Ca^2+^ concentration quantified with the Arsenazo III assay. At mildly acidic pH, metallo-chromogen Arsenazo III combines with calcium to form a colored complex, which absorbs at 650 nm proportionally to the amount of Ca^2+^ in the sample. The Arsenazo III reagent (0.2 mM Arsenanzo III prepared in a 0.1 M imidazole buffer, pH 6.8) was directly added to 20 μL of sample aliquots, mixed vigorously with a vortex mixer for 10 s before adding to a 96-well plate to measure the absorbance at 650 nm. Final Ca^2+^ concentration was determined through comparison to a standard curve made with known standards of CaCl_2(aq)_ prepared from 0 to 10 mM.

### Rheological Measurements

Rheological measurements were conducted using a strain-controlled ARES rheometer (TA Instruments) fitted with a 25 mm cone-and-plate steel geometry, maintaining a gap height of 0.25 ± 0.05 mm. To assess the effects of ionic cross-linking on the viscosity and modulus of the polymer samples, 300 μL of polymer solution was placed on the steel plate, followed by an equal volume of gelling solution pipetted on top and mixed thoroughly. The samples were mixed thoroughly using a 1 mL syringe and allowed to gel for 15 min. Following gelation, the linear viscoelastic region (LVR) was determined for each sample through a dynamic strain sweep from 0.01 to 100% strain at a fixed frequency of 1 Hz. Following the determination of LVR for these cross-linked gels, a dynamic frequency sweep was performed at a fixed strain of 0.5%, well within the LVR of all samples, from 1 to 100 rad/s. Thixotropy was assessed using stepwise applications of high and low strains, where high strains were 400%, well outside the LVR, and low strains were 0.01% well within the LVR. Each was allowed to run for 60 s, and the strain was manually switched. The plates were never opened, and the transition was relatively fast.

### Scanning Electron Microscopy (SEM)

SEM stubs were prepared with conductive carbon tape. For the deposited samples, equal volumes of the polymer and 40 mM calcium chloride solution were applied to the stub. Samples were allowed to gel and were subsequently submerged in liquid nitrogen until completely frozen (∼30 s), and lyophilized. For sprayed samples, polymer and gel solutions were mixed in equal volumes in an Eppendorf tube and sprayed onto the stub. Samples were allowed to set and were subsequently lyophilized. Samples were then analyzed using a Zeiss Sigma VP field-emission scanning electron microscope.

### Cryogenic Transmission Electron Microscopy (CryoTEM)

300-mesh copper grids with lacey carbon film (Electron Microscopy Sciences, Hatfield, PA, USA) were glow-discharged for 30 s in a PELCO easiGlow system (Ted Pella, Inc., Redding, CA, USA) prior to use. Samples were prepared at 0.1 wt % PA concentration prior to vitrification. 7 μL of sample solutions were transferred to the plasma-cleaned 300-mesh copper grids with lacey carbon support and plunge-frozen using a Vitrobot Mark IV (FEI) vitrification robot. Samples were blotted at room temperature with 95–100% humidity and plunged frozen into liquid ethane. Samples were transferred into a liquid nitrogen bath and placed into a Gatan 626 cryoholder through a cryo-transfer stage. Cryo-TEM was performed using a liquid nitrogen cooled JEOL 1230 TEM working at a 100 kV accelerating voltage. Images were acquired using a Gatan 831 CCD camera.

### Adhesion Testing

Adhesion testing was performed based on adjustments made to ASTM F2258 and D4541 using a Mark-10 EasyMESUR Test (Model F105) equipped with a 25 N load cell, as has been previously reported.^11^ Each hydrogel sample had a final volume of 0.3 mL and was used after a thorough mixing of polymer and gel solutions. A tailored pull-off test was performed between two glass substrates with an applied force of 0.16 N resulting from the weight of the dolly, and a pull-off rate of 13 mm min^–1^. To conduct the test, the bottom of an aluminum dolly was modified with a glass coverslip adhered using a commercially available cyanoacrylate adhesive. A glass microscope slide coated with bacon fat was used as the bottom contact, which was adhered to a 3D-printed shape created to fit the bottom clamp of the test instrument. The maximum force was recorded as the force necessary to separate the adhered joint. The use of bacon fat created a fatty substrate base more mimetic to the application of these materials.

### Spray Analysis

To analyze the spray coverage abilities of these samples, Spot-On paper (Innoquest Inc.) was used, and the spray volume was fixed so that 1 mL was used per 80 cm^2^ of the card. Spray was applied from ∼10 cm away, and the force on the syringe was applied manually by the operator. Polymer solution and gel solution were prepared separately, and equal volumes were mixed in an Eppendorf tube vigorously, allowed to gel for 15 min, and then drawn up in a syringe, and subsequently sprayed. Images were acquired following spray, and ImageJ thresholding was applied to differentiate wet Spot-On paper (blue) from dry (yellow), allowing determination of percent area coverage.

### Tissue Simulation for Spray or Injection

To determine the applicability of these materials as sprayable or injectable therapeutics, model systems were employed through the use of chicken drumsticks, which act as a substrate for spray or injection of the polymer. The polymer solution was gelled using gelling solution, and 2 μL of blue food coloring was incorporated. Samples were then drawn and applied either through an 18 G needle or through a syringe spray nozzle.

### Cell Culture Maintenance

Human embryonic kidney cells (HEK 293) were cultured in Dulbecco’s Modified Eagle's Medium (DMEM), which was supplemented with 10% fetal bovine serum (FBS) and 1% Penicillin–Streptomycin. Cells were cultured at 37 °C and 5% CO_2_ and used when the confluence in the flask reached 80%.

### Cytotoxicity

Cells were harvested from culture flasks by using a trypsin–EDTA solution, which was subsequently diluted with enriched DMEM, followed by centrifugation. The resulting cell pellet was resuspended in enriched DMEM and diluted to a concentration of 1 × 10^5^ cells/mL, confirmed using trypan blue staining and an Invitrogen Countess Cell Counter. The supramolecular polymer was then gelled in a 96-well plate in triplicate to a final concentration of 0.25, 0.5, or 1.0 wt % with 20 mM CaCl_2_. Cells were then gently applied to the gels and allowed to incubate.

The cells were incubated at 37 °C in a 5% CO_2_ atmosphere for 48 or 72 h. A spontaneous control was included, wherein cell culture media was replaced with ultrapure filtered water only. Following the exposure, a lactate dehydrogenase (LDH) assay was conducted according to the manufacturer’s instructions for the Invitrogen CyQUANT kit, and percent cytotoxicity was calculated using eqs 1 and 2.

$$\mathrm{LDH}\;\mathrm{Activity}=A_{\mathrm{sample},490}-A_{\mathrm{sample},600}$$ 1

$$\mathrm{Cytotoxicity}\;(\%)=\left(\frac{\mathrm{LDH}\;{\mathrm{Activity}}_{\mathrm{sample}}-\mathrm{LDH}\;{\mathrm{Activity}}_{\mathrm{spon}\tan\mathrm{eous}}}{\mathrm{LDH}\;{\mathrm{Activity}}_{\mathrm{Max}\mathrm{Lysis}}-\mathrm{LDH}\;{\mathrm{Activity}}_{\mathrm{spon}\tan\mathrm{eous}}}\right)\times 100$$ 2

### Cell Imaging

To visualize the impact on cell viability, Live/Dead imaging was also performed by using the ThermoFisher Live/Dead Cell Imaging kit. Cells were cultured as described above but plated in a glass bottom, black-welled, 96-well plate. After 48 h, 50 μL of supernatant was removed from each well, and 50 μL of Live/Dead stain was added. Cells were incubated at room temperature for 15 min prior to imaging on a Leica STELLARIS STED Super-Resolution Confocal Microscope.

### Cell Viability Following Spray Delivery

Viability of HEK 293 cells following spray delivery was assessed following the protocol described previously by Wood et al.^81^ Briefly, HEK293 cells were harvested and prepared at a concentration of 2 × 10^5^ cells/mL in Ringer’s buffered solution and mixed at equal volume with either the Ringer’s buffer or PA supramolecular polymer hydrogel (1 wt %) to achieve a final cell concentration of 1 × 10^5^ cells/mL and PA concentration of 0.5 wt %. Samples were sprayed from a 1 mL syringe using a syringe spray nozzle, and viability following incubation was assessed using trypan blue and manually counted using a hemocytometer, with time points for assessment prior to spray compared to 5 min, 24, 48, and 72 h post-spray. Three aliquots of each suspension were counted, resulting in a total of three values for each sample, completed in triplicate. Mean cell count and standard deviation were then calculated compared to controls.

## Results and Discussion

### PA Supramolecular Polymer Characterization

A single PA monomer was utilized for this study to isolate the impacts of cell loading on the self-assembly and mechanical properties of the resultant gels. The PA monomer consists of a hydrophobic palmitic acid tail, which promotes hydrophobic collapse of the core, a sequence of three alanine and three valine residues which support intermolecular cohesion through hydrogen bonding, and three glutamic acids to improve aqueous solubility (Figure 1a). The supramolecular polymerization of this PA monomer is well established, resulting in high-aspect-ratio nanofibers, which entangle into three-dimensional scaffolds, capable of supporting cells as a scaffold for tissue regeneration.^82–84^ The monomer was synthesized by automated solid-phase peptide synthesis, and purity was confirmed by high-resolution liquid chromatography-mass spectrometry (LC-MS, Figure S1).

**Figure 1:**
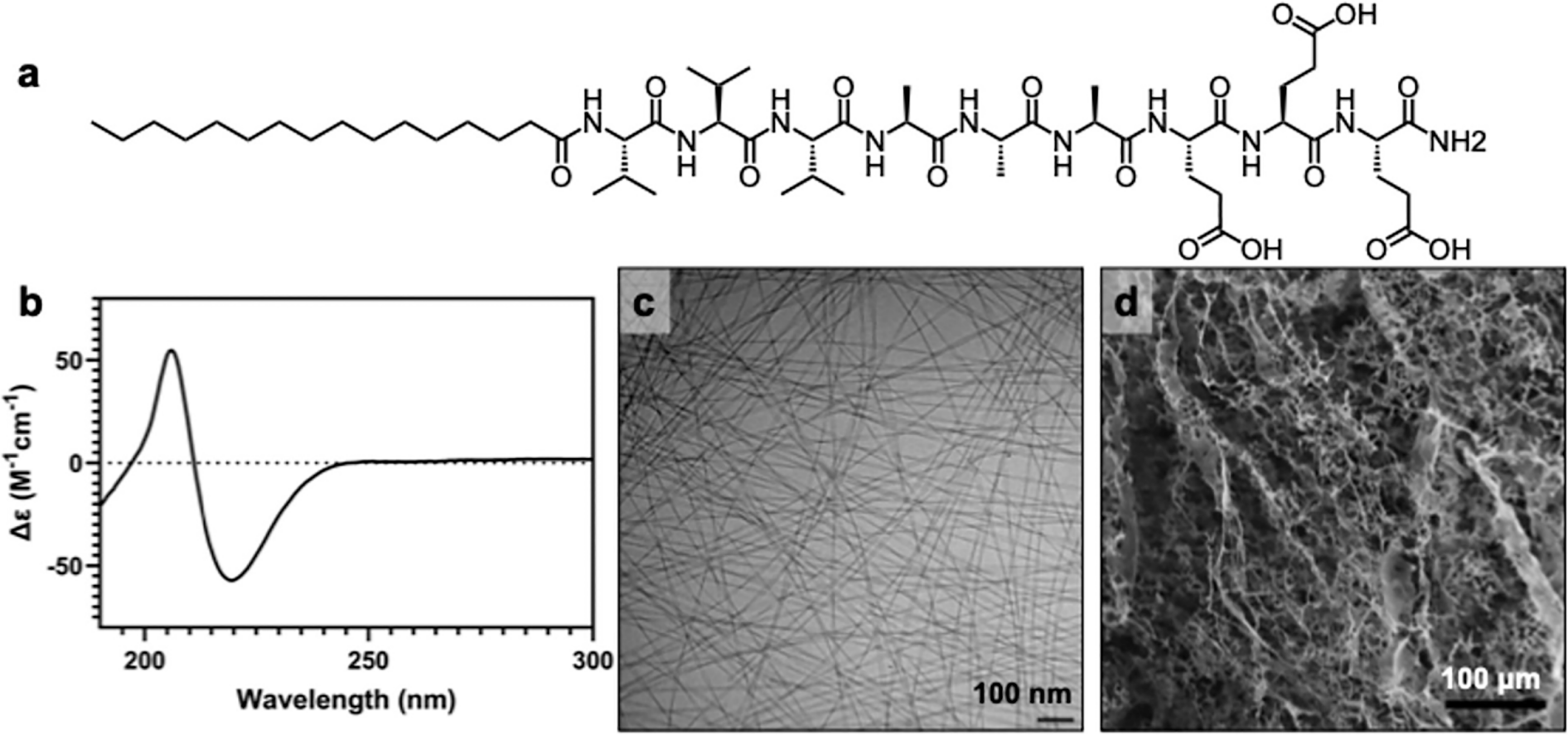
Nanofibrous PA supramolecular polymers. (a) Molecular structure of the PA monomer investigated in this study and (b) circular dichroism of the PA supramolecular polymer. (c) Cryogenic transmission electron microscopy of the PA supramolecular polymer nanofibers and (d) SEM of PA supramolecular polymer scaffold.

To assess the internal cohesion between PA monomers, circular dichroism (CD) was used to confirm the presence of strong beta-sheet intermolecular hydrogen bonding, as observed by the characteristic CD minima at 218 nm and maxima at 195 nm (Figure 1b). Cryogenic transmission electron microscopy (CryoTEM) analysis confirmed the successful supramolecular polymerization of the monomers into high-aspect-ratio elongated nanofibers with uniform widths (Figure 1c). Complementary SEM following ionic cross-linking with calcium ions provided insight into the porosity and topography of the PA supramolecular polymer hydrogel, revealing a well-defined nanofibrous network highly reminiscent of the extracellular matrix (ECM), and well suited for cell infiltration and scaffolding (Figure 1d).

### Design of Experiments (DoE) Approach

A two paired two-factor, three-level full factorial DoE with three replicates was implemented to systematically interrogate the design space and evaluate potential interactions between matrix concentration and cell concentration in both a covalently cross-linked alginate hydrogel and a supramolecular PA nanofiber hydrogel. While conventional three-level factorial designs typically employ evenly spaced factor levels (low, mid, and high) to ensure orthogonality and maximize statistical efficiency, the present study deliberately employed nonequidistant levels to more accurately capture the experimentally relevant performance boundaries of the materials at translationally relevant conditions, employing a DoE approach to our study. In the PA nanofiber hydrogel, concentrations below 0.25 wt % yielded irregular, weakly structured gels with liquid-like rheological behavior, precluding meaningful assessment of cell–matrix interactions. Conversely, concentrations exceeding 1 wt % imposed disproportionately high material demands with minimal incremental gains. Thus, the selected factor levels were intentionally biased toward the concentration range in which both systems exhibited structurally stable, physiologically relevant behavior, enabling higher-resolution evaluation of material–cell interactions in the most informative region of the design space (Table 1). To adjust for this lack of orthogonality, factor levels were coded to improve statistical orthogonality using eq 3.

$$x_{\mathrm{coded}}=\frac{x-x_{\mathrm{center}}}{\mathrm{half}-\mathrm{range}}$$ 3

**Table 1:** Experimental Design and Values and Levels of Independent Variables and Responses

determinants	code	lower level	coded low input	middle level	coded middle input	upper level	coded upper level
polymer conc. (wt %)	X_1_	0.25	–1	0.5	–0.33	1	1
cell conc. (cells/mL)	X_2_	1 × 10^5^	–1	1 × 10^6^	0.0526	2 × 10^6^	1

This normalization to dimensionless coded variables is a requisite to mitigate scale disparities, enhance numerical conditioning of the regression matrix, and preserve interpretability of estimated effects in the absence of strict orthogonality.

### Rheological Stability

The entangled PA nanofibers can be ionically cross-linked to tune both the viscosity and stiffness of the hydrogel produced through chelation of the gamma-carboxyl groups of glutamic acid on adjacent nanofibers, with a divalent cation such as calcium to produce an ionically cross-linked supramolecular polymer matrix. In the gelation of the supramolecular hydrogel, the calcium acts as an electrostatic bridge between nanofibers, modulating interfiber interactions and overall networking of the system.^82,85,86^ To evaluate the specific effects of cell–hydrogel interactions on the mechanical properties of supramolecular hydrogels, we maintained a constant calcium chloride (CaCl_2_) concentration of 20 mM during hydrogel formation across PA and alginate systems. This concentration was selected to achieve partial saturation of glutamic acid residues across low and high supramolecular polymer concentrations, ensuring that none of the hydrogels we planned to investigate were fully cross-linked. To confirm this, a frequency sweep was performed to determine viscosity and stiffness as a result of increasing CaCl_2_ concentrations at different polymer concentrations, where it was determined that the gel properties were not significantly altered above a threshold of approximately 50 mM of CaCl_2_ (Figures S2 and S3). As a result, it was determined that working at a constant 20 mM CaCl_2_ concentration would allow us to compare the impacts of cell loading in robust hydrogels. This approach provided for the isolation of cell-specific contributions to the hydrogel’s mechanical behavior. Calcium quantification assays revealed an initial release of ∼10 mM Ca^2+^ from the supramolecular hydrogels regardless of PA concentration, consistent with a loosely bound fraction readily exchanged to the sink solution (Figure S4). When the release sink was regularly replenished with fresh buffer to approximate infinite dilution conditions, a slower, concentration-dependent release profile emerged in which lower PA concentrations liberated more calcium initially, reflecting reduced densities of the chelating capability. After approximately 5 h, release plateaued at a level maintained for days, indicative of a stably coordinated fraction that persists under physiological conditions and is sufficient to sustain network integrity, highlighting similarities in loadings across all hydrogels (Figure S5).

To assess the effect of cell loading on hydrogel viscosity, a frequency sweep at 0.5% strain was performed for each condition in our DoE. Both supramolecular polymer and covalent alginate controls exhibited shear-thinning behavior, consistent with disruption of ionic cross-links under shear (Figure S6). Increasing supramolecular polymer concentration shifted viscosity upward across all shear rates, reflecting denser network formation, and cell inclusion did not significantly alter this profile. In contrast, alginate hydrogels displayed a marked dependence on cell loading, with viscosity decreasing at higher cell concentrations, particularly in higher weight-percent formulations (Figure S6). This reduction likely arises from displacement of the matrix by cells, which limits cross-linking and the ability of the covalent backbone to accommodate this displacement. The dynamic, fiber-bridged architecture of the supramolecular system accommodates cell incorporation without compromising network integrity, in contrast to the more rigid covalent alginate network.^87,88^ The egg-box model is traditionally used to describe how divalent metal ions, such as Ca^2^^+^, coordinate with adjacent G-blocks to form a three-dimensional gel network with alginate.^89–91^ The rigidness of the covalent backbone, along with the highly specific binding of the G-blocks, produces a less dynamic hydrogel when compared to that of the PA supramolecular polymer hydrogel, as seen by the rheological response of the gels.

To evaluate the structural integrity of the cell-laden PA supramolecular polymer hydrogels, we conducted further rheological analysis to determine the LVR. Notably, variations in cell incorporation did not influence the crossover point or the plateau modulus of the biomaterials significantly (Figure S7), suggesting that cellular inclusion does not compromise the mechanical stability of the dynamic hydrogel network. Subsequent measurements for impacts on modulus were performed via frequency sweeps at 0.5% strain, ensuring that measurements remained within the LVR (Figure S8). PA supramolecular polymer hydrogels exhibited minimal changes in crossover point, plateau modulus, or frequency-dependent behavior with increasing cell concentration, indicating that their dynamic, fiber-bridged architecture accommodates cellular incorporation without loss of stiffness. In contrast, we observed a pronounced decrease in modulus across all formulations of the ionically cross-linked alginate hydrogels, with the effect amplified at higher polymer concentrations. We hypothesized this weakening of the hydrogel network occurs from geometric disruption of the Ca^2^^+^-mediated cross-links within the rigid polysaccharide backbone, as densely packed cells displace the matrix and hinder junction formation (Figures 2 and S8). At 1 Hz, no supramolecular polymer hydrogel exhibited a statistically significant stiffness loss relative to the cell-free control, suggesting that the fibrous scaffold can dynamically rearrange to preserve material integrity. Collectively, these results underscore the greater resilience of dynamic supramolecular networks to high filler content compared to that of their covalent counterparts. We attribute the PA’s resilience to its dynamic, electrostatically bridged supramolecular fiber network, which undergoes a rapid, reversible reorganization under stress, enabling recovery after local disruptions caused by cell incorporation. In contrast, the ionically cross-linked structure of the covalent alginate polymer relies on discrete, rigid G-block junction zones; displacement by cells disrupts these junctions irreversibly, likely reducing the overall cross-link density. This microstructural sensitivity explains the pronounced modulus loss in alginate gels and supports a refined design principle: under high filler loadings, cells function as mechanical defects within the hydrogel network, and overall material performance is governed by the network’s ability to redistribute stress and tolerate defect density at the bulk scale. While both systems rely on reversible ionic interactions, supramolecular hydrogels that combine redundant cross-linking with a dynamically reconfigurable fiber backbone exhibit superior defect tolerance and mechanical recovery, enabling retention of bulk properties under high-shear processing conditions that are not accessible in ionically cross-linked covalent polymer networks.

**Figure 2:**
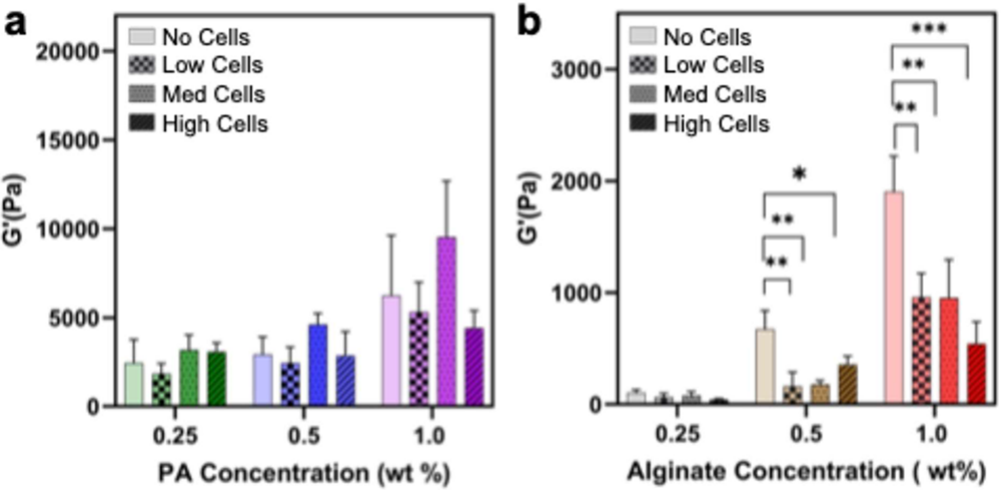
Rheological analysis of stiffness as a function of matrix concentration and cell loading. Dynamic frequency sweeps from 1 to 100 rad/s of cell-laden hydrogels were conducted to determine the impact of cell loading on stiffness, with the 1 Hz data point isolated for analysis. (a) *G*′ of supramolecular polymer hydrogels at 1 Hz with increasing cell concentration. (b) *G*′ of alginate gels at 1 Hz with increasing cell concentration. Data displayed as mean ± standard deviation, minimum of *n* = 3 independent runs. * Denotes *p* < 0.05. Statistical significance was determined using a one-way ANOVA, with a post hoc Tukey test for means comparison.

### Hydrogel Adhesion Assessment

In tissue engineering, scaffold adhesion to the wound bed is a critical design constraint, influencing integration, mechanical stability, ease of application, and ultimately regenerative outcomes. We hypothesized that the incorporation of cells into all hydrogel networks would disrupt the network integrity and reduce adhesion to a substrate. Despite the addition of high filler concentrations, the network structure and mechanical properties of the gels remained largely unchanged. Adhesion testing, therefore, served as an orthogonal measure of gel robustness, reflecting the preserved polymer packing and network integrity. To assess adhesion, a glass substrate was first coated with bacon fat (i.e., fat-coated) to mimic the lipid-rich surface chemistry of deep dermal or full-thickness skin injuries, and an adhesion pull-off test administered with the cell-laden hydrogels (Figure 3a,b). To ensure the sensitivity of force head to our application, we first validated by comparing the PA supramolecular polymer on a fat-coated surface pre- and post-cross-linking with calcium ions. As the concentration of uncross-linked polymer increased, interactions with the substrate also increased, leading to higher adhesion forces. Upon ionic cross-linking, the carboxyl groups of glutamic acid residues were occupied within the hydrogel network, thereby reducing their availability for interaction with the substrate surface. While the adhesion of PA nanofibers is typically expected to be dominated by hydrogen bonding between surface carboxylic acids and hydroxylated substrates, we observe enhanced adhesion to fat-coated surfaces only when the carboxylates are free. Cross-linking led to a negligible change in the adhesion force dependent on cell concentration or weight percent solid content of the matrix, but a mitigated response compared to the uncross-linked polymer (Figure 3c). Prior to data collection, the system’s limitations and interactions with the fatty substrate were investigated to ensure robust experimental design and ensure any impacts from coating the substrate or cross-linking did not influence results, with a negligible impact from fat-coating the surface observed following cross-linking of the materials (Figure S9). Interestingly, despite significant differences in their structural compositions, both hydrogels exhibited remarkably similar adhesion forces to the fatty substrate (Figure 3d–f). Statistically significant differences in the adhesion of PA and alginate matrices to the fat-coated surface were observed only at the highest cell loading in the low and medium weight percent formulations, as well as in the neat 0.5 wt % matrix comparison. This observation underscores the complexity of hydrogel adhesion mechanisms, which are influenced not only by surface chemistry but also by the hydrogel’s microstructure. These findings highlight the multifactorial nature of hydrogel adhesion, where surface chemistry alone is insufficient to predict the performance. Instead, the interplay between network architecture, cross-linking density, and swelling behavior governs the available interfacial binding motifs and their capacity to form stable adhesive contacts.

**Figure 3:**
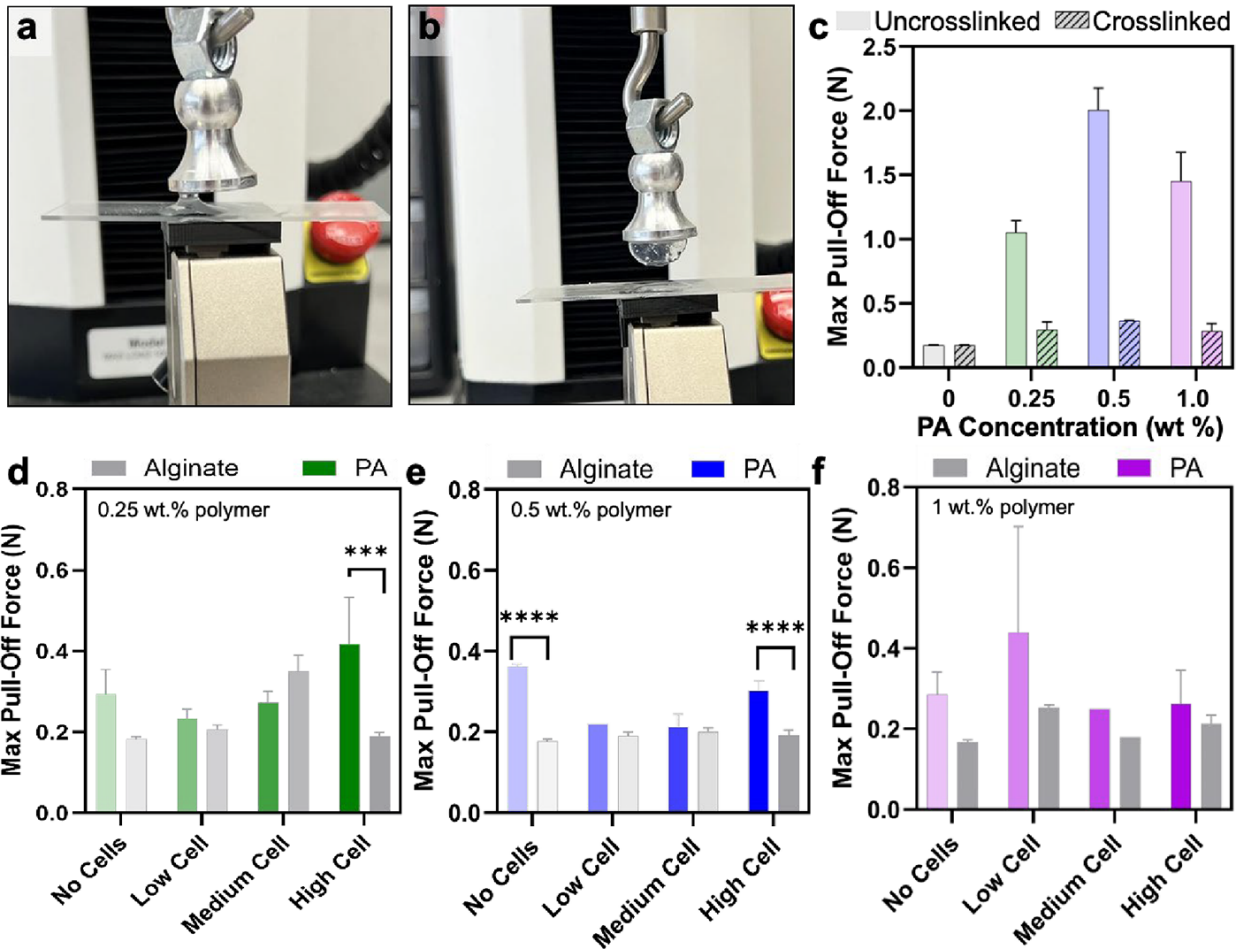
Characterization of adhesion to a fat-coated substrate. Image of pull-off test being conducted with (a) uncross-linked and (b) cross-linked supramolecular polymer on a fat-coated glass surface. (c) Adhesion force from pull-off test of uncross-linked and cross-linked PA supramolecular polymers. Adhesion force from pull-off test of (d) 0.25 wt % cell-laden hydrogels, (e) 0.5 wt % cell-laden hydrogels, and (f) 1.0 wt % cell-laden hydrogels. * Denotes *p* < 0.05 for comparison between matched alginate and PA supramolecular polymer data points, determined in GraphPad Prism using a one-way ANOVA with a post hoc Tukey test for means comparison.

### Spray Analysis and Demonstration of Hydrogel Delivery

The application of cell-laden hydrogels via spray delivery offers a promising approach for treating complex wounds, facilitating enhanced cell localization at the wound bed, and potentially accelerating the healing process. To evaluate the efficacy and precision of this delivery method for our biomaterials, we used Spot-On paper, a water-sensitive substrate primarily used in agricultural settings to assess spray deposition, in conjunction with image analysis techniques. Upon wetting, the Spot-On paper changes from yellow to blue in regions where the hydrogel made contact, providing a visual representation of the deposition area. This colorimetric change enabled the quantification of spray coverage and uniformity, ensuring a consistent application across a simulated wound site. The fixed deposition area and spray volume parameters allowed for controlled experiments, facilitating reproducibility and reliability in assessing the hydrogel’s performance. Although both the covalent and supramolecular hydrogels were ionically cross-linked and possessed shear-thinning profiles, only the supramolecular polymer effectively sprayed through the nozzle with consistently high surface area coverage (Figure 4a,b). Independent of polymer concentration, successful spray coverage was achieved, even at the highest cell incorporation assessed in our DoE (Figure 4c).

**Figure 4:**
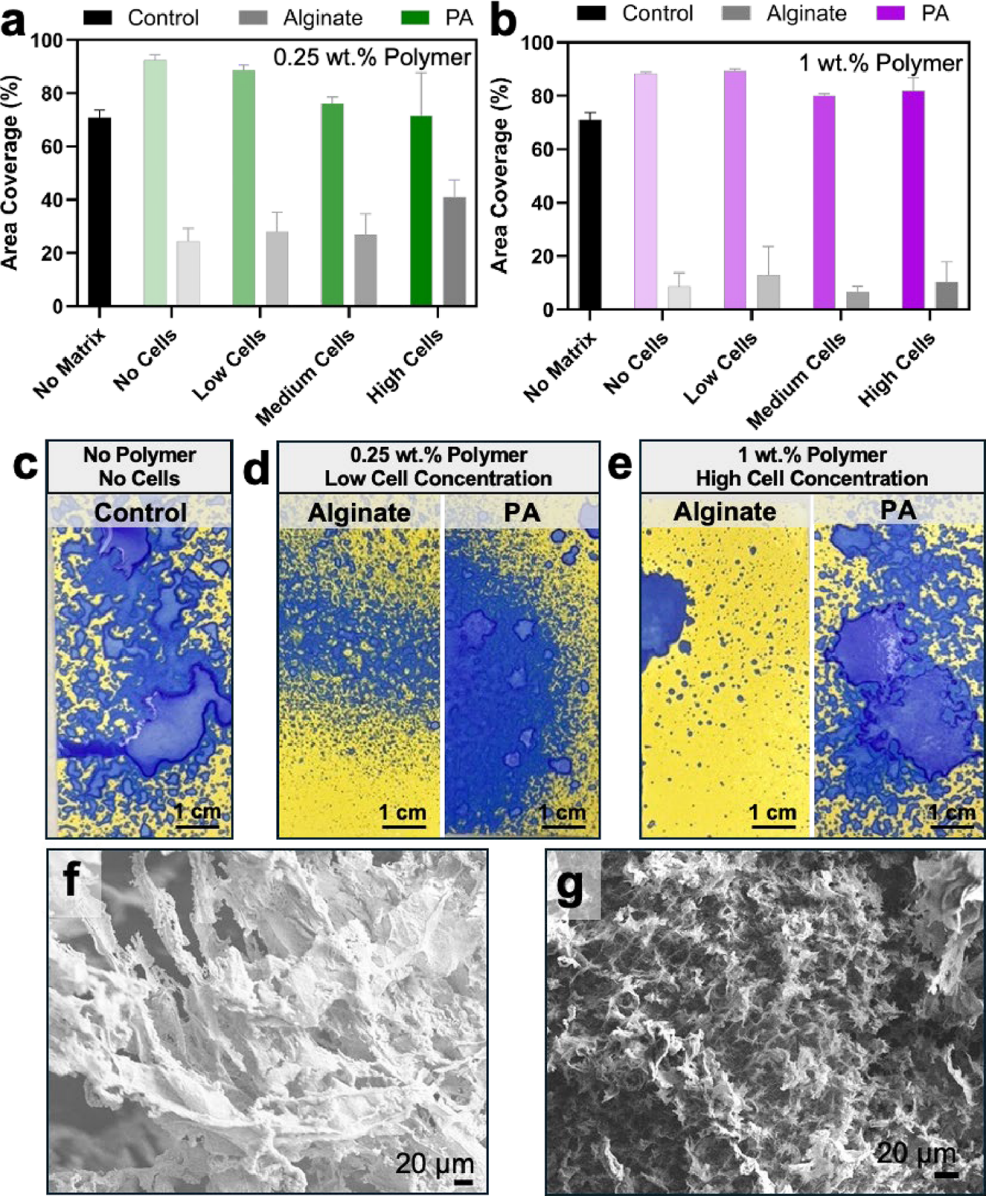
Characterization of sprayability of cell-laden gels. Area coverage by sprayed cell-laden hydrogels at (a) low polymer concentration and (b) high polymer concentration. Representative images of Spot-On paper (yellow= dry, blue = wet) depicting the spray of supramolecular and covalent hydrogels of (c) control, (d) low polymer, low cell loading condition, and (e) high polymer, high cell loading condition. Representative scanning electron microscopy images of the ionically cross-linked supramolecular polymer (f) before and (g) after spray delivery.

The supramolecular polymer hydrogel effectively transitions from gel to solution through the high shear of spray and rapidly reforms the hydrogel following deposition. The ionic cross-linked hydrogel is disrupted by the high shear through the nozzle resulting in supramolecular polymer disentanglement. Independent of matrix concentration or filler incorporation, the supramolecular hydrogel could cover >80% of the designated substrate area (Figure 4a,b) for a controlled volume of hydrogel. The alginate hydrogel was sprayable at lower polymer concentrations, with increasing substrate coverage observed as the cell concentration increased (Figure 4a). However, as polymer concentration increased for the alginate hydrogels, spray area coverage was significantly reduced, especially compared to the equivalent conditions in the PA supramolecular polymer hydrogel (Figure S10). Fundamentally, cell incorporation disrupted cross-link formation and network integrity, resulting in increased liquid character of the gel, which was also observed by rheology.

At low alginate concentrations, partial disruption of the ionic network lowered yield stress and improved sprayability, but higher polymer content established dense cross-link domains between proximal chains, producing a rigid gel that extruded rather than atomized (Figure S11). While ionic chelation between alginate guluronate blocks is essential for gelation, the overall rigidity of the egg-box network is fundamentally dictated by the stiff polysaccharide backbone, which limits chain flexibility and enhances mechanical resistance. The preorganized, semi-rigid chain geometry constrains segmental motion, amplifying the structural reinforcement imparted by the cross-links.

Scanning electron microscopy was used to examine how spray delivery affects the topography and porosity of the PA supramolecular hydrogels. Compared to ionic cross-linked controls, sprayed hydrogels exhibited thinner scaffold features and reduced banding, consistent with shear-induced disruption of the ionic cross-links. The dynamic, supramolecular backbone allowed the network to deform under high shear during spraying and then rapidly reform upon cessation of flow, re-establishing hydrogel architecture. Despite these transient disruptions, each sample retained scaffold-like features with interconnected pores suitable for cellular migration, demonstrating the potential of PA supramolecular polymer hydrogels as sprayable tissue scaffolds (Figure 4f,g).

The PA supramolecular polymer hydrogel retains its gel architecture and mechanical integrity, even at high cell-loading levels, reflecting the resilience of its dynamic, noncovalent network. To illustrate its practical utility, the hydrogel was delivered to a chicken drumstick via subcutaneous injection and topical spray delivery (Figure 5a,b). Upon injection, the material remained precisely localized, while the sprayed hydrogel adhered to the tissue surface, resisting both mechanical agitation and submersion in water (Supporting Information, Videos S1 and S2).

**Figure 5:**
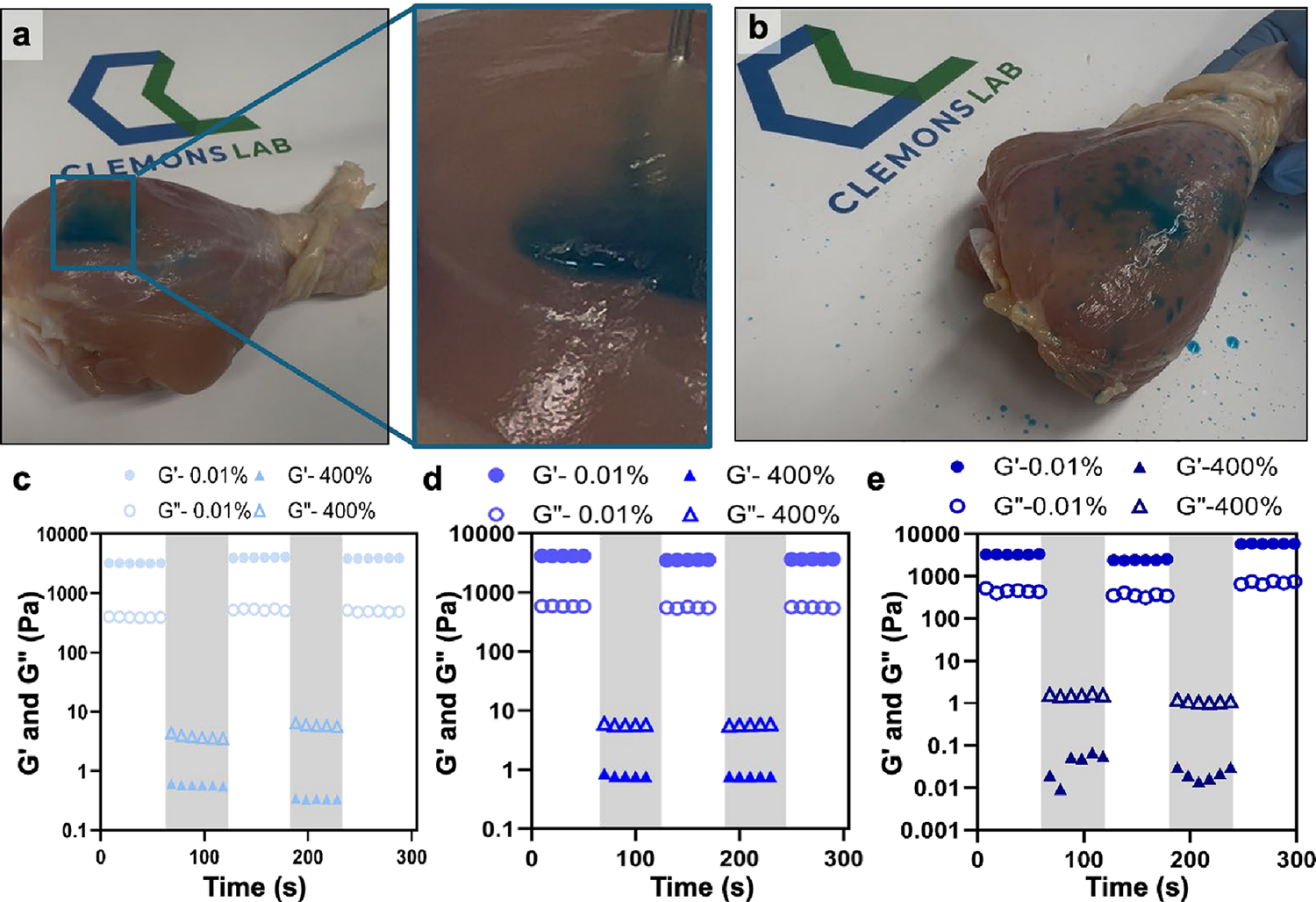
Simulated application of high-shear delivery of supramolecular polymer hydrogels. (a) Injection of 0.5 wt % PA supramolecular polymer hydrogel into a chicken drumstick, showing injectability and localization. (b) Spray of 0.5 wt % PA supramolecular polymer hydrogel through a nozzle showing sprayability and regelation on the tissue surface. Three-step oscillation methods for thixotropy analysis of 0.5 wt % PA supramolecular polymer hydrogels with (c) no cell loading, (d) at low cell loading, and (e) at high cell loading. PA supramolecular polymer hydrogels in panels (a) and (b) loaded with blue food coloring to enhance visualization. Logo reproduced with permission from the author; © 2026 Clemons Lab LLC.

Thixotropic recovery of the PA hydrogel was quantified by using a three-step oscillatory protocol. A low-strain within the LVR was alternated with a high-strain well beyond the LVR to induce significant deformation, followed by a return to low strain. Across formulations with no cells, low cells, and high cell loading, the hydrogel exhibited complete recovery, tolerating applied strains up to 400% without compromising mechanical integrity (Figures 5c–e). In contrast, alginate at high matrix concentrations, despite exhibiting thixotropic and shear-thinning behavior (Figure S12), the rigid cross-links and covalent backbone resist uniform shear, especially at the extremely high shear experienced during spray delivery, causing nozzle fouling, and incomplete droplet breakup (Supporting Information, Video S3). This contrast highlights how the supramolecular PA hydrogel uniquely combines high-shear resilience with sprayability, making it suitable for minimally invasive delivery.

Cytocompatibility of the PA hydrogels was assessed with both quantification of lactate dehydrogenase (LDH) release and live cell confocal fluorescence microscopy. Cell seeding density was constrained by confluency limitations over time, precluding the use of higher initial cell concentrations to directly match cell concentrations used in the DoE assessment of hydrogel mechanical properties. Cell-based cytocompatibility assays were conducted to assess PA supramolecular polymer hydrogel-associated cytotoxicity in static gels following spray delivery (Figure 6). The low cytotoxicity of alginate-based systems is well established and matches what was observed in the PA systems.^38,92^ Following 48 h of incubation, viable cells were observed actively migrating through the PA supramolecular polymer hydrogel (Figure 6a–c). Notably, cell migration was evident across all hydrogel concentrations tested, with no discernible dependence on polymer hydrogel concentration or significant cytotoxicity observed (Figure 6d). Cells were detected at multiple focal planes, indicating three-dimensional translocation through the network, and were observed adhering to the well-plate surface post-migration, similar to the no-hydrogel control (Figure S13a). No significant increase in cytotoxicity was observed after 72 h of incubation, highlighting the stability and cytocompatibility of the PA supramolecular polymer hydrogels (Figure S13b). We further assessed cell viability following spray delivery, where the addition of the PA hydrogel had no negative impact on the outcome of cell viability over 72 h of incubation (Figure 6e). Notably, incorporation of the PA supramolecular polymer hydrogel enhanced post-spray cell retention and adhesion (Figure 6f), as cells sprayed into a Petri dish and subsequently inverted remained within the dish, demonstrating strong substrate adhesion relevant to future tissue regeneration applications. Collectively, these results confirm the high cytocompatibility of the PA supramolecular hydrogels and their ability to preserve cell survival and function following high-shear delivery.

**Figure 6:**
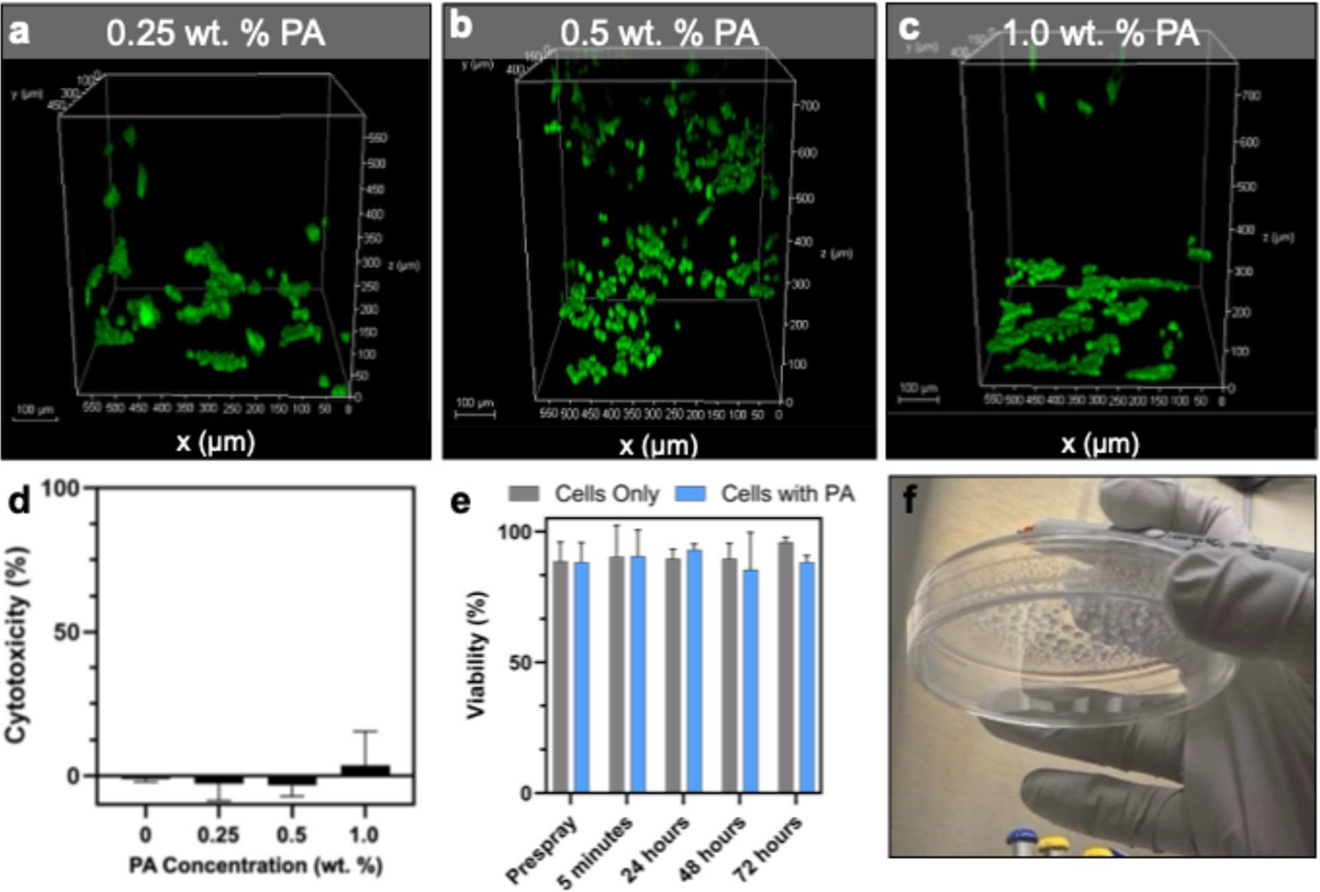
Cytocompatibility assessment of the PA supramolecular polymer hydrogel. Confocal microscopy of human embryonic kidney (HEK 293) cells seeded on top of hydrogels and following incubation visualized using Calcein-am for live cell imaging of (a) 0.25 wt %, (b) 0.5 wt %, and (c) 1.0 wt % of the PA supramolecular polymer hydrogel. (d) Lactate dehydrogenase assay (LDH) of the PA supramolecular polymer hydrogel following 48 h incubation with HEK 293 cells. (e) Cell viability of HEK 293 cells assessed following spray delivery with the PA supramolecular polymer hydrogel (0.5 wt %, blue) compared to the cell only control (gray). (f) Image of cells with the PA supramolecular polymer hydrogel following spray delivery into a Petri dish and inverted demonstrating hydrogel adhesion.

### Multilevel Factorial Design

Two full factorial designs were initially planned to understand the functions and limitations of cell loading within the PA supramolecular polymer hydrogel compared to the covalent polymer alginate hydrogel, wherein a quality-by-design approach was applied to evaluate gel formulations containing varying polymer and cell filler levels. We sought to understand the interaction and influence of these parameters on dependent response variables (*Y*_1_–*Y*_3_) that impact the application of cell-laden hydrogels. The filler and polymer levels were informed by similar studies with covalent, ionic polymer gels, and we expected to obtain some level of maximum cell loading where significant loss of mechanical properties would be observed. Excitingly, no cell filler level within our range interacted negatively with the PA supramolecular polymer hydrogels. The main effects plots highlighted the impact of polymer concentration on mechanical properties, but not at the cost of application-based response variables. At increasing concentrations of PA supramolecular polymer, higher viscosity and stiffness were achievable, due to greater entanglement and intramolecular cross-linking (Figure 7a–c). Further, when compared to no cell controls conducted outside of the DoE framework, there was no significant decrease in hydrogel performance with any amount of filler incorporation. In contrast, alginate-based hydrogels exhibited a pronounced decrease in mechanical properties with an increasing filler content. Specifically, the addition of fillers to alginate hydrogels led to a significant reduction in viscosity and stiffness at each concentration assessed, with the extent of performance loss correlating with the concentration of alginate and the concentration of incorporated cells (Figure 7d–f). This decline in mechanical properties was attributed to disruptions in the packing and cross-linking of the alginate network. This is further supported by interaction plots that highlight shifts in properties dependent on cell concentration in the alginate hydrogel (Figure S14).

**Figure 7:**
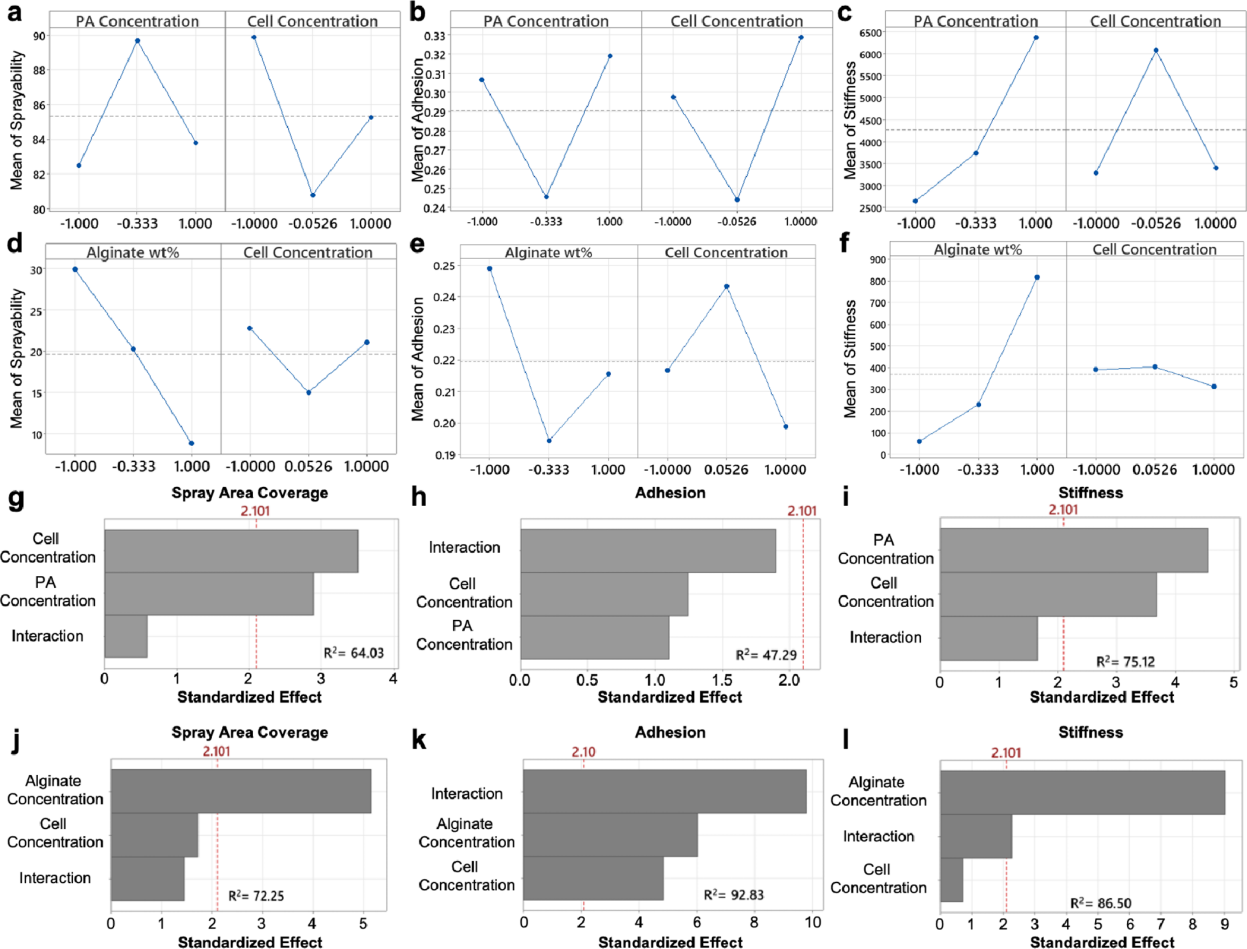
DoE response of full factorial design for both systems. Main effects plots for the PA network response variables: (a) sprayability, (b) adhesion, and (c) stiffness. Main effects plots for the covalent alginate network response variables: d) sprayability, (e) adhesion, and (f) stiffness. Pareto charts on factors impact in the PA system on response variables: (g) sprayability, (h) adhesion, and (i) stiffness analysis. Pareto charts on factors impact in the covalent alginate system on response variables: (j) sprayability, (k) adhesion, and (l) stiffness analysis. Significance is denoted by bars crossing the red dashed line. Statistical analysis was performed using Minitab Statistical software, with significance set at α = 0.05.

Statistical analysis of the standardized effects revealed that while both cell and supramolecular polymer concentrations independently influenced stiffness, area coverage, and adhesion, their interaction term consistently fell below the significance threshold (α = 0.05, Figure 7). This indicates that the combined effect of cell and PA supramolecular polymer hydrogel does not produce a synergistic or antagonistic response in the measured outcomes. The decoupled nature of these variables suggests that the cellular and matrix contributions to material and biological responses can be tuned independently. Further, the only measured response for the PA supramolecular polymer hydrogel with significance in the standard effects plot is the spray area coverage. However, none of these factors reduced spray area coverage below that of the control spray outside the DoE, suggesting this may be an artifact of the Spot-On substrate or the pooling and droplet coalescent behavior (Figure 7g–i). In contrast, the alginate system has a significant dependence on the interaction term for both stiffness and adhesion response variables, highlighting the destructive impact of increasing filler concentration on material properties in the covalent polymer networks (Figure 7j–l).

The alginate-based polymer demonstrated an amplified response and greater sensitivity to cell loading and polymer concentration, resulting in pronounced property changes and high coefficient determination. In contrast, the PA supramolecular polymer hydrogel exhibited subtler responses and lower *R* ^2^, reflecting a lower signal-to-noise ratio where experimental variability dominates. This disparity highlights intrinsic differences in material behavior: the dynamic, reversible nature of supramolecular assemblies complicates modeling, whereas the alginate system behaves predictably and is well captured by the chosen factors. Consequently, cell loading and polymer concentration significantly explain the variability in the alginate system but less so for the PA supramolecular polymer within the same design space. Additionally, while calcium concentration was held constant to limit the dimensionality of the DoE space, increasing polymer content necessarily alters the effective cross-link density in both hydrogel systems, and this factor should be considered when interpreting absolute moduli across formulations or in future studies. The use of a DoE approach allowed us to better understand the entire design space and implications of cell loading and polymer concentration on final material properties, wherein the dynamic nature of the supramolecular backbone provides an avenue for higher cell incorporation with maintained material properties.

## Conclusions

This study provides a structure–function framework for designing PA supramolecular polymer hydrogels capable of successful cell delivery through the high shear associated with injection or spray for regenerative medicine applications. By employing a full factorial DoE approach, we elucidated the independent and combined effects of the polymer concentration and cell loading on hydrogel mechanical integrity, adhesion, and sprayability. Notably, the PA supramolecular polymer hydrogels maintained rheological stability and structural cohesion even under high cell loading, in stark contrast to alginate-based controls, which suffered significant mechanical property loss for equivalent cell loadings. The dynamic, non-covalent beta-sheet-driven self-assembly of the PA supramolecular polymer hydrogels, coupled with non-covalent ionic cross-linking, affords inherent adaptability under shear, enabling both injectability and sprayability without sacrificing performance. The ability of these materials to rapidly reform a hydrogel following high shear delivery, adhere to soft tissue substrates, and recover their structure following repeated mechanical perturbation, positioning them as ideal candidates for next-generation, minimally invasive regenerative therapies. Rheological and adhesion analyses demonstrate that cellular incorporation minimally impacts mechanical integrity or substrate binding, while SEM confirms rapid network reformation following deformation, highly mimetic of the natural ECM. In contrast, ionically cross-linked alginate exhibits modulus loss and shear sensitivity due to disruption of rigid junction zones. These results highlight a generalizable design principle: reversible, supramolecular cross-linking enables sprayable, cell-laden scaffolds that maintain both structural and functional performance, offering a robust platform for minimally invasive tissue engineering applications. This work underscores the utility of supramolecular design principles in overcoming limitations in the field of biomaterial design for high-shear and minimally invasive delivery regimes.

## Supplementary Material









## Notes

LC-MS trace of PA monomer; concentration and cross-link density impacts on complex viscosity; cross-link density impacts on rheological properties of 1 wt % supramolecular polymer; gelation of supramolecular polymer using calcium chloride and subsequent release characterization; cumulative calcium release by Arzenazo III reagent over 72 hours; rheological analysis of stiffness as a function of matrix concentration and cell loading; determination of LVR of polymer gels; frequency sweep of cell-laden hydrogels with varying wt % matrix and cell loading; determination of impacting on fat coating and cross-linking for adhesion pull-off test; complete area coverage data of alginate and PA sprayed onto Spot-On paper; challenges spraying alginate hydrogel through nozzle; thixotropy of high concentration alginate (1 wt %) highlighting material recovery following extreme deformation; cell viability of PA supramolecular polymer scaffolds; and interaction plots for significant effects on results (PDF)
Injection delivery of PA hydrogel (MP4)
Spray delivery of PA hydrogel (MP4)
Spray attempt of alginate hydrogel (MP4)
