# Supplementary material for: Cell-Laden Supramolecular and Covalent Polymer Hydrogels for High-Shear Delivery: A Design of Experiments Approach
Source: Chem Mater. 2026 Feb 16;38(5):2357–71. doi: 10.1021/acs.chemmater.5c03073 (PMC12980631; doi:10.1021/acs.chemmater.5c03073)
Supplement: Supplementary file 1 [file cm5c03073_si_001.pdf]

Cell-Laden Supramolecular and Covalent Polymer Hydrogels for High-Shear Delivery: A Design of Experiments Approach

Penelope E. Jankoski,[1#] Jessica Shrestha,[1#] Windfield S. Swetman,[1] Harrison Livingston,[1] Jamie Sorrell,[1,2] and Tristan D. Clemons.[1*]

1 School of Polymer Science and Engineering, University of Southern Mississippi, Hattiesburg, MS, 39406, USA.

2 Sumrall High School, Sumrall, MS, 39402, USA.

# Authors contributed equally to the work

*Correspondence should be addressed to T.D.C (email: Tristan.clemons@usm.edu)

Supporting Information

Table of Contents

Figure S1. LC-MS trace of PA monomer. 2

Figure S2. Concentration and crosslink density impacts on complex viscosity. 3

Figure S3. Crosslink density impacts on rheological properties of 1 wt% supramolecular polymer. 3

Figure S4. Gelation of supramolecular polymer using calcium chloride and subsequent release characterization. 4

Figure S5. Cumulative calcium release by Arzenazo III reagent over 72 hours. 4

Figure S6. Rheological analysis of stiffness as a function of matrix concentration and cell loading 5

Figure S7. Determination of LVR of polymer gels. 6

Figure S8. Frequency sweep of cell-laden hydrogels with varying wt% matrix and cell loading 7

Figure S9. Determination of impacting on larding and crosslinking on adhesion pull-off test.. 8

Figure S10. Complete area coverage data of alginate and PA sprayed onto Spot-On Paper. 8

Figure S11. Challenges spraying alginate hydrogel through nozzle 9

Figure S12. Thixotropy of high concentration alginate (1 wt%) highlighting material recovery following extreme deformation 9

Figure S13. Cell Viability of PA supramolecular polymer scaffolds. 10

Figure 14. Interaction plots for significant effects on results. 11

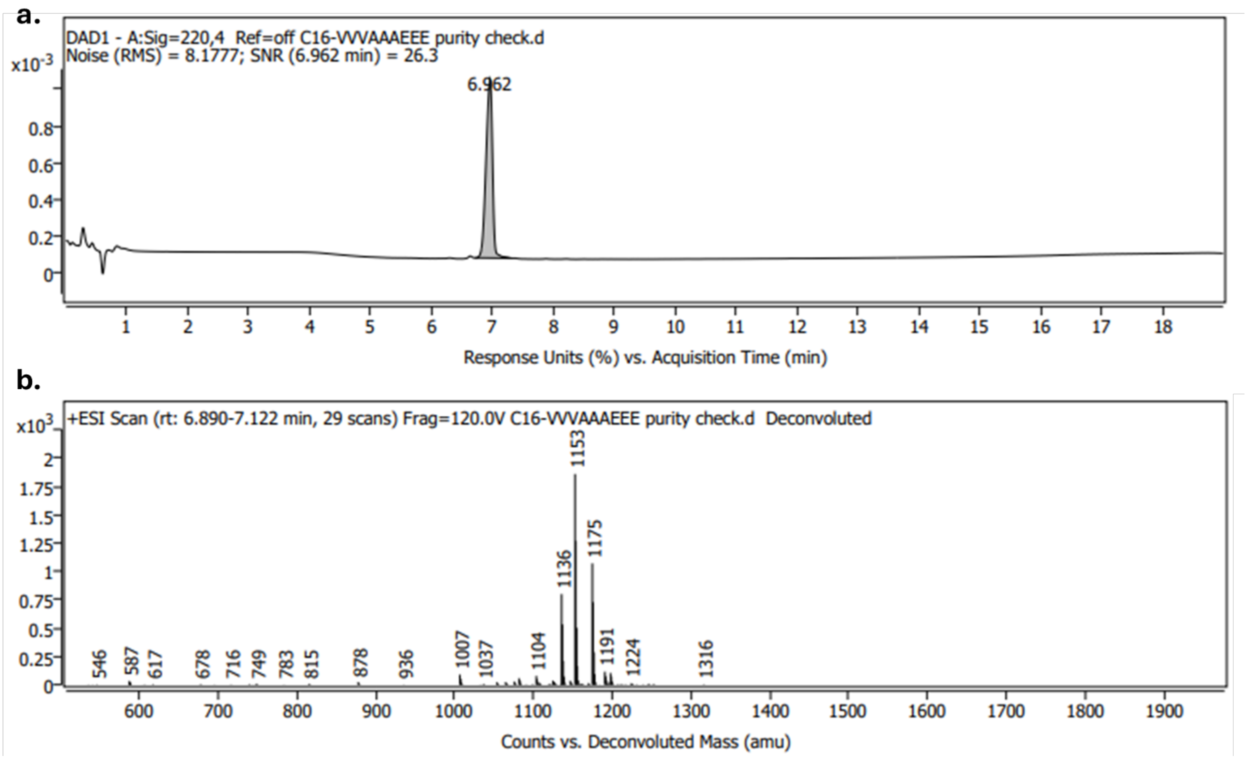

Figure S1. LC-MS trace of PA monomer. a) LC-MS trace of control PA [PA] = 1 mg/mL, loading solvent; H2O with 0.1% NH4OH (v/v), eluent; H2O-CH3CN gradient containing 0.1% HCOOH (v/v), column; Phenomenex Gemini 5 µm C18 110 Å LC column 150 x 1 mm and ESI-mass spectra corresponding to elution time 6.89 – 7.12 min.

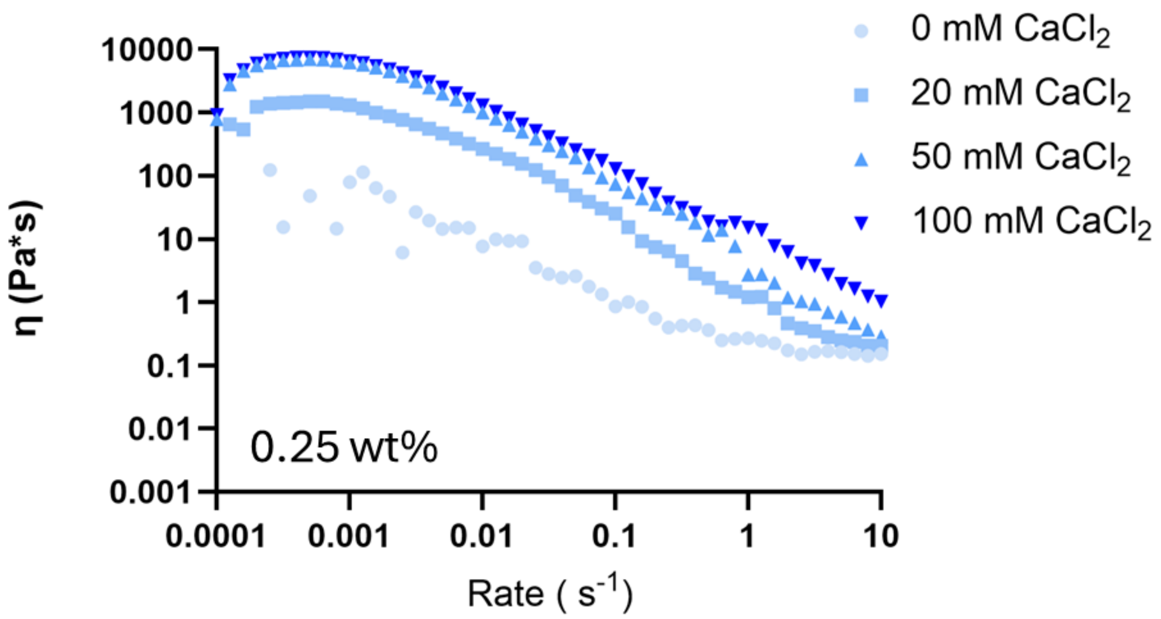

Figure S2. Concentration and crosslink density impacts on complex viscosity. 0.25 wt% supramolecular polymer gelled by increasing concentrations of $CaCl_2$ to determine saturation.

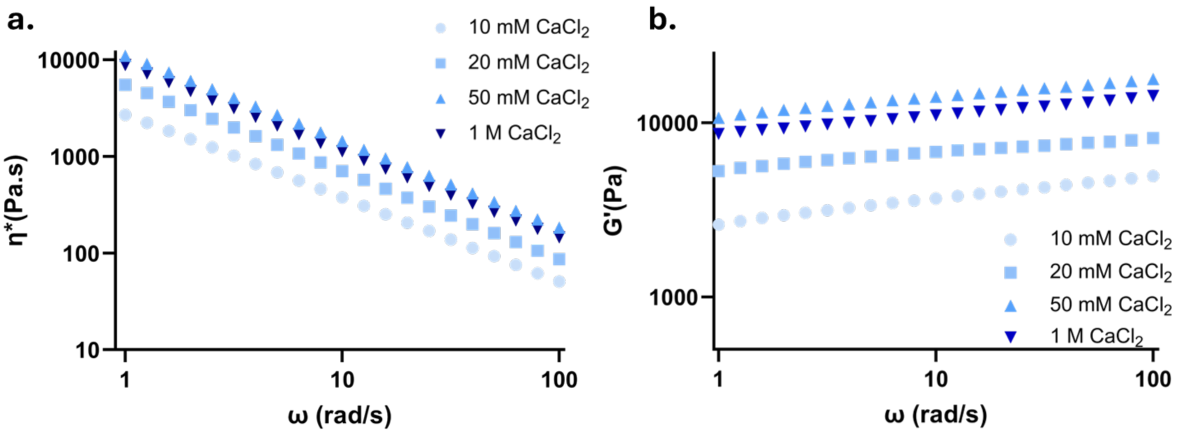

Figure S3. Crosslink density impacts on rheological properties of 1 wt% supramolecular polymer. a) Impacts of increasing $CaCl_2$ concentration on stiffness of resulting ionic gel from 1 wt% supramolecular polymer b) Impacts of increasing $CaCl_2$ concentration on viscosity of resulting ionic gel from 1 wt% supramolecular polymer.

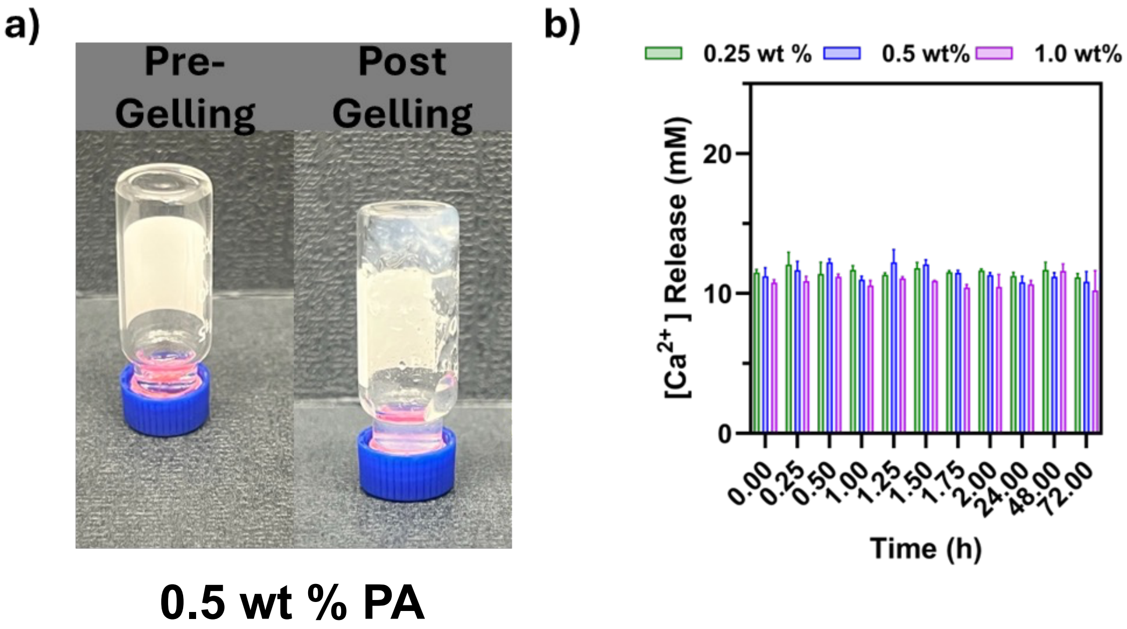

Figure S4. Gelation of supramolecular polymer using calcium chloride and subsequent release characterization. a) Ionic gelation of 0.5 wt % PA in inverted HPLC vial pre and post $CaCl_2$ addition, b) No significant change in calcium release was observed from PA hydrogels over 72 hours when release sink was held constant.

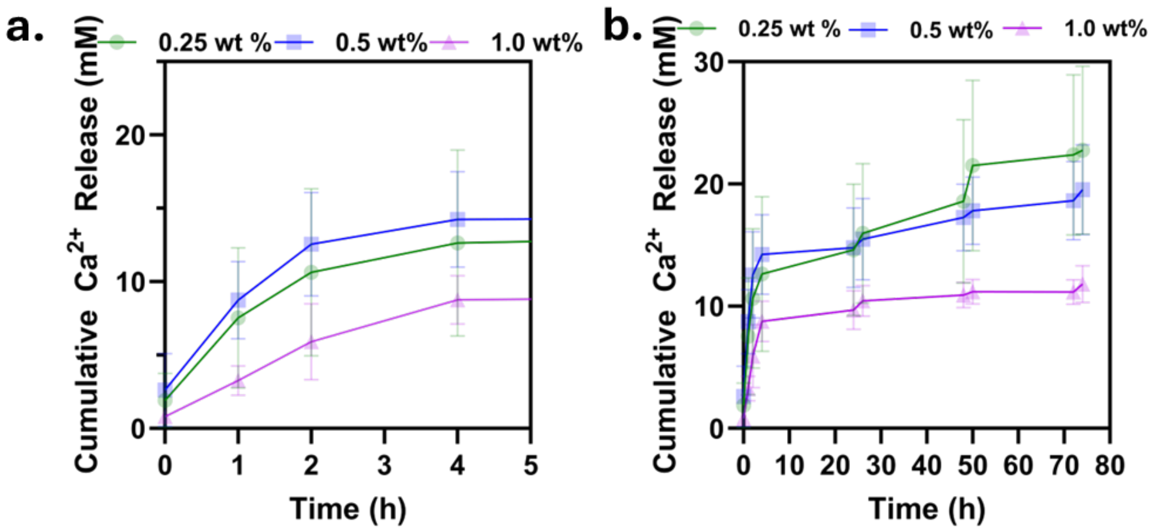

Figure S5. Cumulative calcium release by Arzenazo III reagent over 72 hours. Release profile highlights an a) initial burst release in the first 5h, and b) a plateau in the calcium release regardless of matrix concentration at later timepoints.

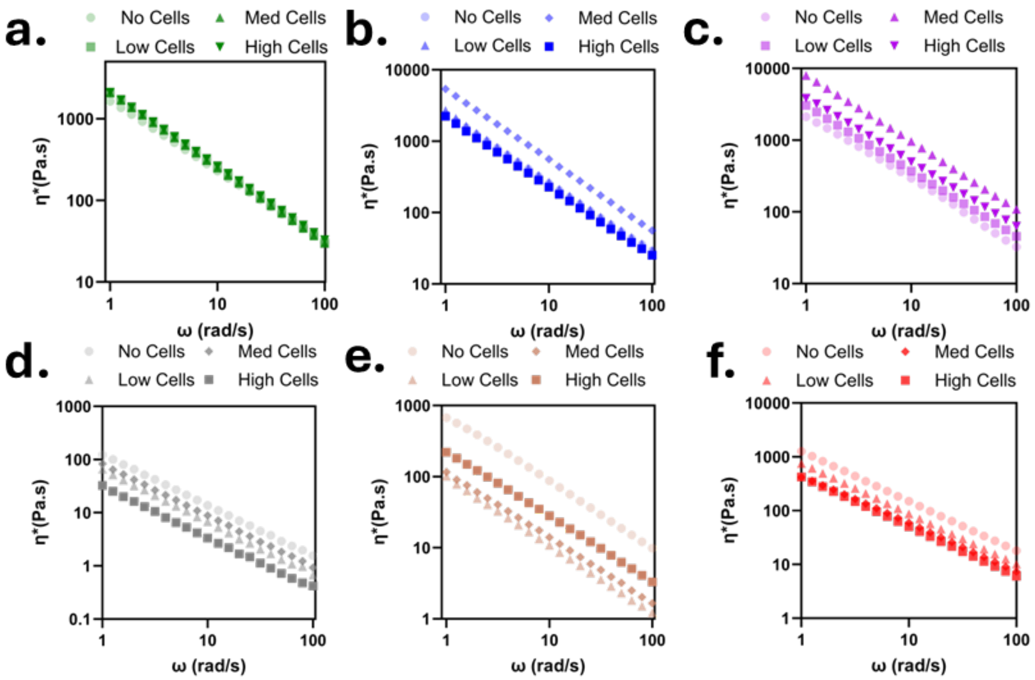

Figure S6. Rheological analysis of stiffness as a function of matrix concentration and cell loading. Dynamic frequency sweeps from 1 to 100 rad/s of cell-laden hydrogels to determine the impact of cell loading on viscosity. In all graphs, no cells is the lightest and highest cell loading is the darkest color a) 0.25 wt % PA, b) 0.5 wt% PA, c) 1 wt % PA, d) 0.25 wt% alginate gel, e) 0.5 wt% alginate, f) 1 wt% alginate. Data displayed is a representative sample from n=3 independent runs.

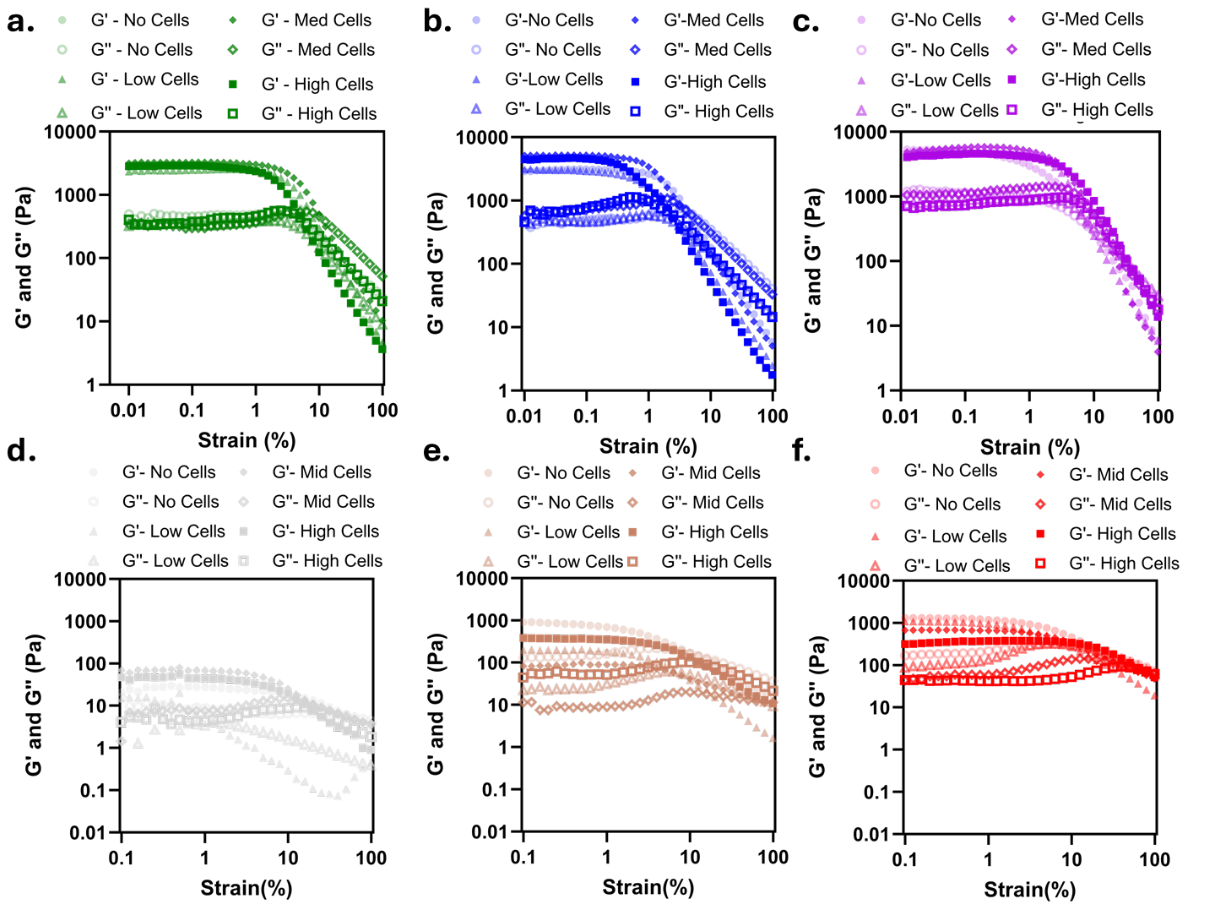

Figure S7. Determination of LVR of polymer gels. a) [PA] = 0.25 wt% with increasing cell loading, b) [PA] = 0.5 wt% with increasing cell loading, and c) [PA] = 1.0 wt% with increasing cell loading, d) [Alginate]= 0.25 wt% with increasing cell loading, e) [Alginate]= 0.5 wt% with increasing cell loading, f) [Alginate]= 1.0 wt% with increasing cell loading.

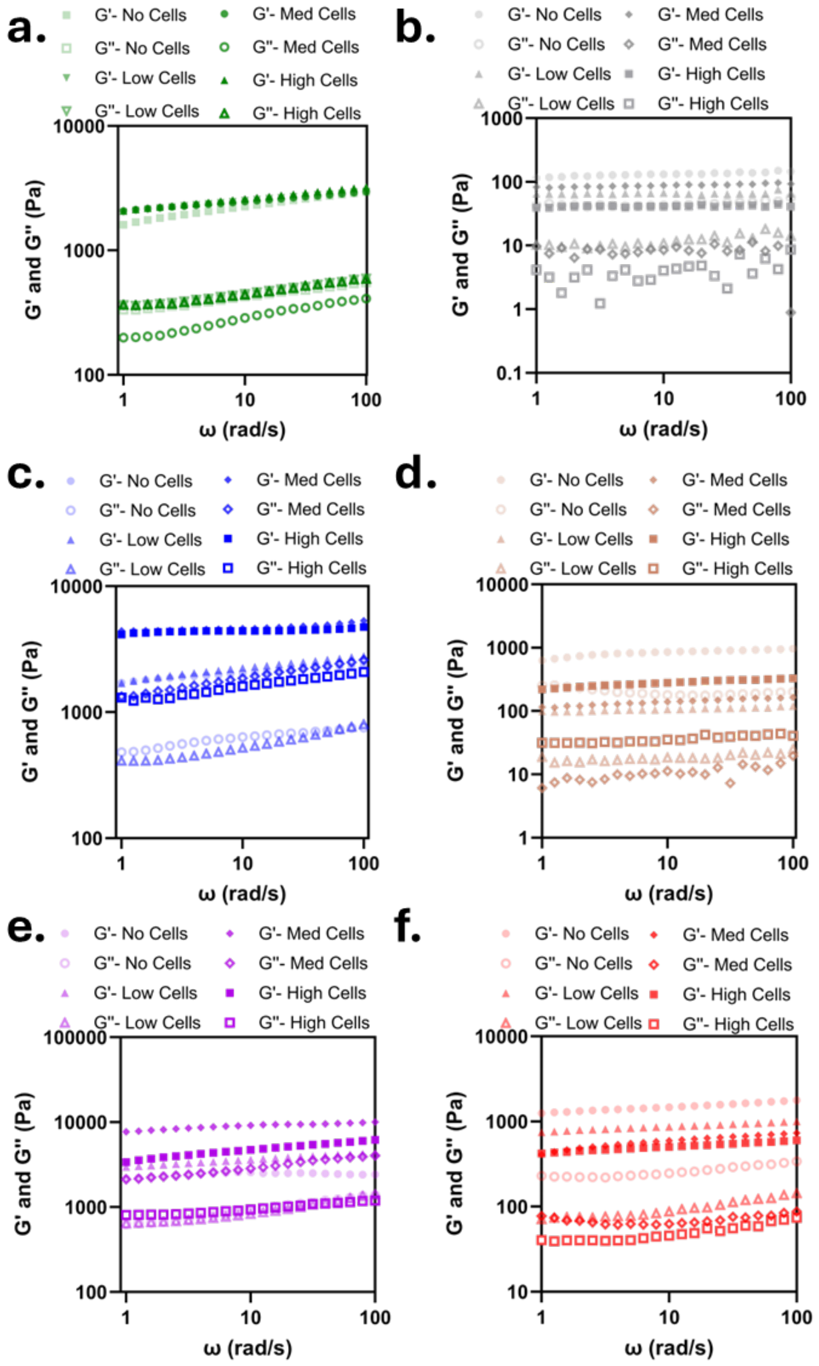

Figure S8. Frequency sweep of cell-laden hydrogels with varying wt% matrix and cell loading. Dynamic frequency sweeps from 1 to 100 rad/s of cell-laden hydrogels to determine the impact on G' and G'' from various cell loading levels. In all graphs, no cells is the lightest and highest cell loading is the darkest color. Solid legends represent G' and the matched empty symbol is corresponding G''. a) 0.25 wt% PA, b) 0.25 wt% alginate gel, c) 0. 5 wt% PA, d) 0.5 wt% alginate, e) 1wt% PA f) 1 wt% alginate.

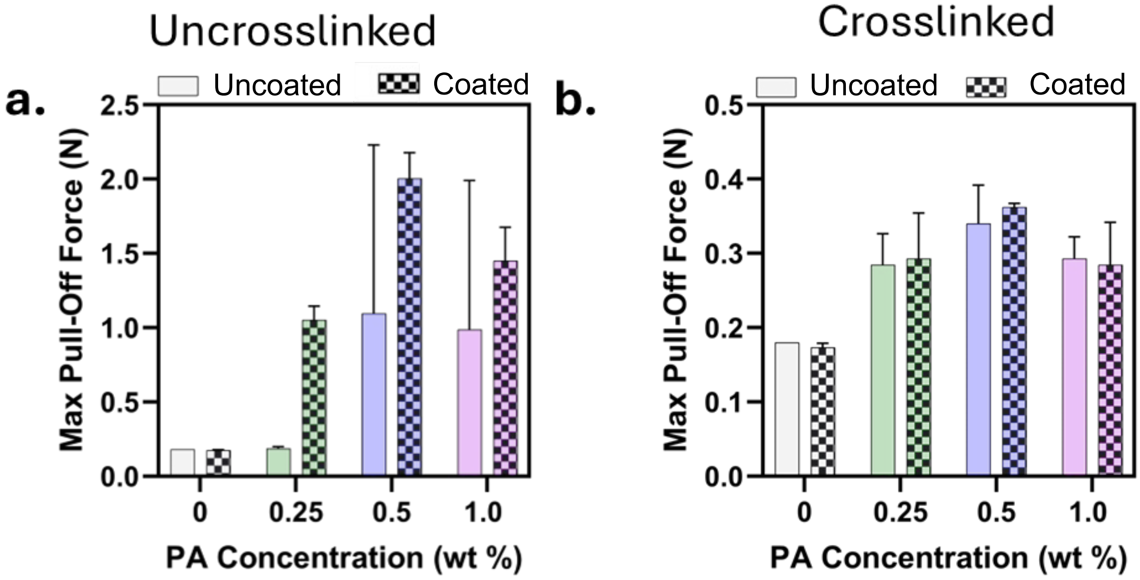

Figure S9. Determination of impacting on larding and crosslinking on adhesion pull-off test. Comparison of a) uncrosslinked and b) crosslinked supramolecular polymers on glass (uncoated) and fat-coated glass substrates.

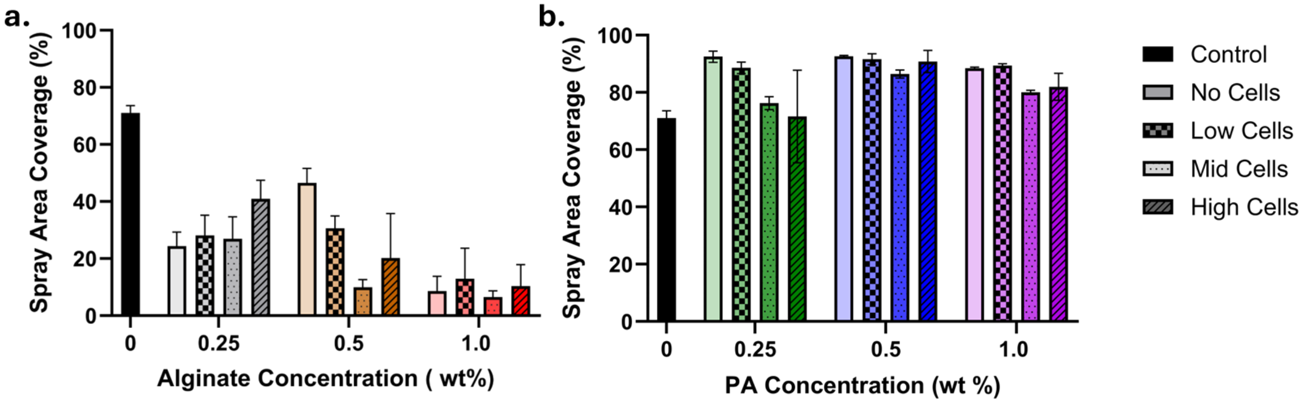

Figure S10. Complete area coverage data of alginate and PA sprayed onto Spot-On Paper. a) Alginate area coverage when a fixed volume was sprayed over a fixed area. b) PA supramolecular polymer area coverage when a fixed volume was sprayed over a fixed area.

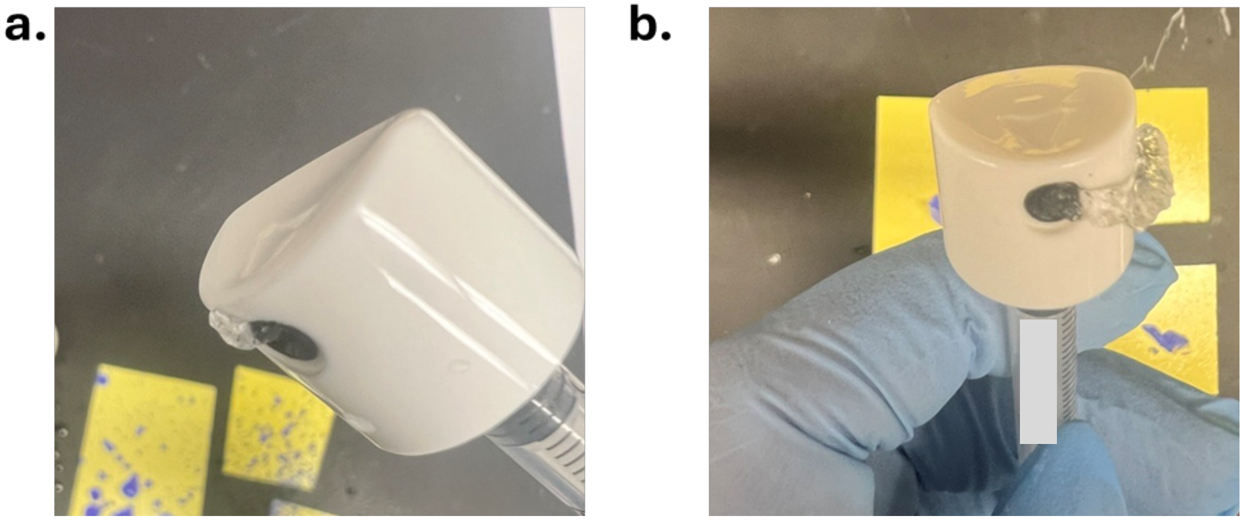

Figure S11. Challenges spraying alginate hydrogel through nozzle. a) 0.25 wt% alginate chunk stuck in nozzle, b) 1 wt % alginate extruding through nozzle.

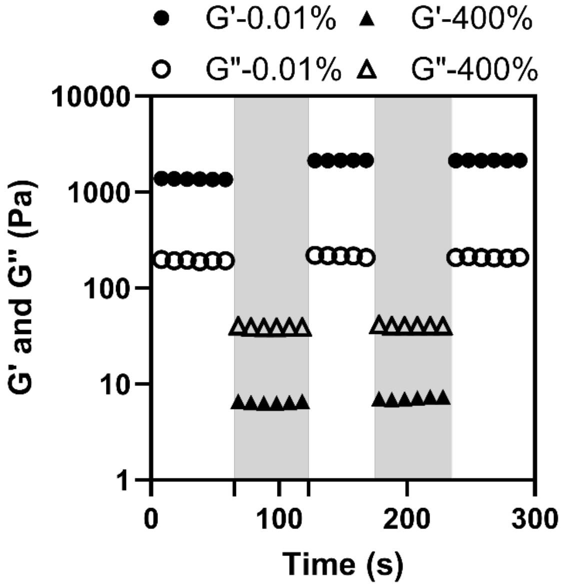

Figure S12. Thixotropy of high concentration alginate (1 wt%) highlighting material recovery following extreme deformation.

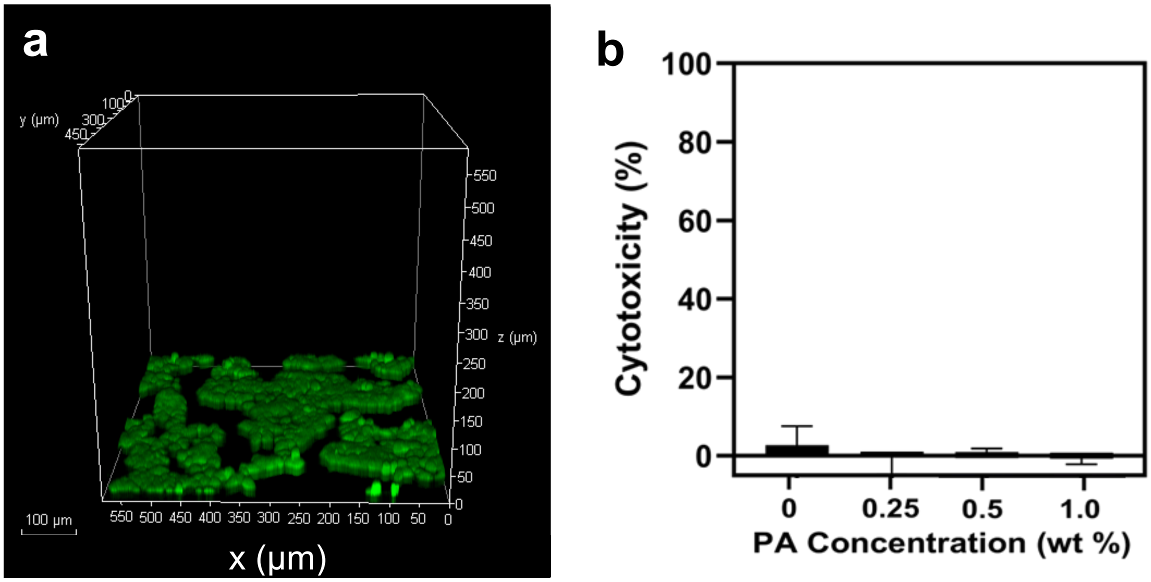

Figure S13. Cell Viability of PA supramolecular polymer scaffolds. a) Confocal microscopy of HEK 293 control cells in the well plate following 48 h of incubation (i.e. no PA hydrogel) and visualized using Calcein-am for live cell imaging. b) Cytotoxicity of HEK 293 cells as assessed by quantification of LDH release following 72-hour incubation with the PA supramolecular polymers. Significance was determined with GraphPad Prism using a One Way ANOVA, with a post hoc Tukey test for means comparison.

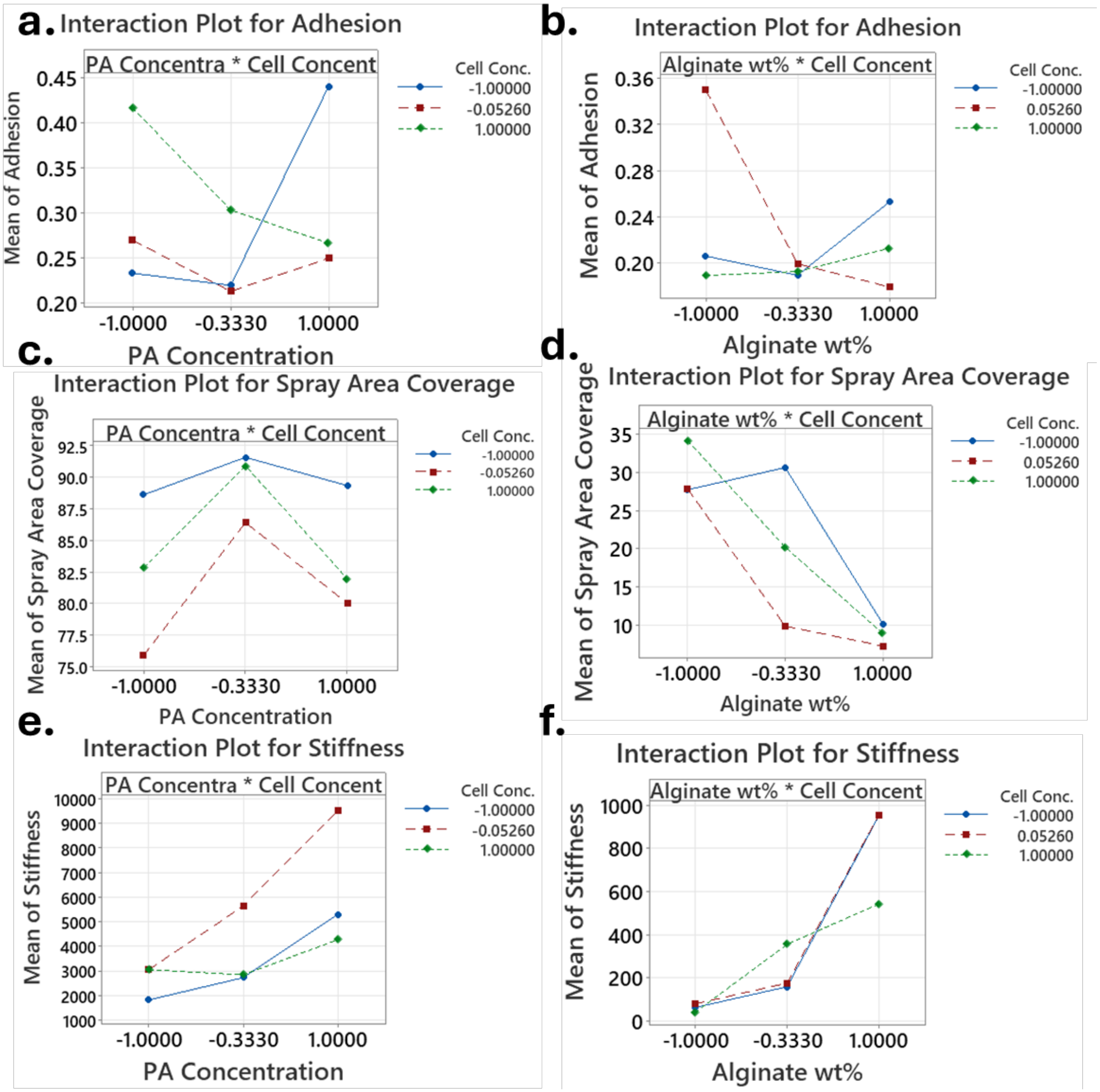

Figure 14. Interaction plots for significant effects on results. a) Interaction plot for adhesion based on PA concentration and cell loading level, with increasing impact on adhesion from low cell loading. b) Interaction plot of alginate on adhesion depends strongly on cell concentration, c) Interaction plot for spray area coverage based on PA concentration and cell loading level, with impact on sprayability from cell loading, with reasonable area coverage from all formulations, d) Interaction plot shows that spray area coverage decreases sharply with increasing alginate concentration for all cell concentrations, with the highest coverage occurring at low alginate content and high cell concentration, e) Interaction plot for stiffness based on PA concentration and cell loading level, with good agreement in low and high cell loading on mechanical stiffness, f) interaction plot shows that stiffness increases dramatically with alginate concentration for all cell concentrations, with the highest stiffness at high alginate and low-to-intermediate cell loading, while high cell loading reduces stiffness.
